# Identifying genetic determinants of outer retinal function in mice using a large-scale gene-targeted screen

**DOI:** 10.1371/journal.pgen.1011886

**Published:** 2025-09-29

**Authors:** Janine M. Wotton, Mark P. Krebs, Riccardo Sangermano, Jessica K. Wong, Cynthia Smith, Amelia M. Willett, Douglas Howell, Abby Jones, Catherine Witmeyer, Jacob P. Lowy, Michael McFarland, Stephan A. Murray, Robert E. Braun, Patsy M. Nishina, Eric A. Pierce, Emily M. Place, Kinga M. Bujakowska, Neal S. Peachey, Jacqueline K. White

**Affiliations:** 1 Center for Biometric Analysis, The Jackson Laboratory, Bar Harbor, Maine, United States of America; 2 Mammalian Genetics, The Jackson Laboratory, Bar Harbor, Maine, United States of America; 3 Ocular Genomics Institute, Massachusetts Eye and Ear, Boston, Massachusetts, United States of America; 4 Pathology Services, The Jackson Laboratory, Sacramento, California, United States of America; 5 Ophthalmic Research, Cole Eye Institute, Cleveland Clinic, Cleveland, Ohio, United States of America; 6 Research Service, VA Northeast Ohio Healthcare System, Cleveland, Ohio, United States of America; 7 Department of Ophthalmology, Cleveland Clinic Lerner College of Medicine of Case Western Reserve University, Cleveland, Ohio, United States of America; National Institute on Deafness and Other Communication Disorders, National Institutes of Health, UNITED STATES OF AMERICA

## Abstract

Electroretinography (ERG) provides a noninvasive functional measure of multiple cell types of the outer retina. We conducted an ERG-based screen of 530 single-gene knockout mouse strains generated as part of the International Mouse Phenotyping Consortium, representing 2.5% of all protein-coding genes, to identify genetic variants affecting retinal function. We identified 30 strains with significantly altered ERG amplitudes. Two of the genes identified, *Cfap418* and *Syne2*, have been previously reported with outer retinal dysfunction, thereby serving as internal controls that validate our screening protocol. Of the remaining 28 genes newly associated with altered retinal function, the majority lacked a contemporaneous histopathology correlate, highlighting the importance of ERG in early detection of functional abnormalities. A rare homozygous missense variant in *FCHSD2*, the human orthologue of one of the 28 genes identified, was found in a patient presenting with retinal degeneration that lacked a molecular diagnosis. This report represents a useful resource for future investigations into the molecular mechanisms driving inherited retinal diseases and demonstrates the power of large-scale ERG screening in identifying novel genetic determinants of retinal function.

## Author summary

Vision depends on the retina, a neural tissue at the back of the eye that converts light into electrical signals. These signals are processed locally and then communicated to the brain. Understanding how the retina is assembled during development, how its health is maintained during a lifetime, and how it contributes to vision at the molecular level will require a complete list of the genes involved in these processes. To expand an already extensive list, we generated 530 mouse strains, each modified to inactivate a distinct gene. We then tested these strains for altered retinal function using electroretinography, which measures electrical signals generated by the retina in response to light. This large-scale approach identified 30 genes that influence retinal function; 28 were not previously associated with this role. Future studies of these genes may advance our understanding of retinal development and maintenance, and the molecular basis of vision. We also identified a rare mutation in the human version of *Fchsd2*, one of the 28 genes, in a patient with inherited retinal degeneration. Large-scale discovery of genes that influence retinal function may benefit efforts to diagnose and treat diseases that impair vision.

## Introduction

The mammalian retina is an exquisitely organized laminar tissue comprised of neurons, glia, epithelia, and blood vessels. Neuronal cell bodies in the retina are organized into three nuclear layers. These layers connect across the outer and inner synaptic layers, where visual signals are processed before being transmitted to the brain via the optic nerve. An epithelial monolayer, the retinal pigment epithelium (RPE) is situated between photoreceptors and the choriocapillaris and plays critical roles in maintaining the sensitivity of rod and cone photoreceptors [1], the former of which can reliably detect a single photon [2]. Single-cell expression studies have provided important insights into the molecules that govern the specialized processes performed by each retinal cell type [3,4]. Critical roles are revealed when mutations result in cell dysfunction and/or anatomical abnormalities, whether in human retinal disease (www.retnet.org) or in naturally occurring animal models [5,6]. Programs involving the use of *N*-ethyl-*N*-nitrosourea-induced random mutagenesis followed by ocular screens have further contributed to the identification of many of the molecules that we now know play critical roles in the mammalian retina [7–14].

The International Mouse Phenotyping Consortium (IMPC) is comprised of 21 international research institutions that use high-throughput, standardized pipelines (www.mousephenotype.org/impress) to comprehensively phenotype single-gene knockout mouse strains, with the goal of understanding the function of all protein-coding genes. Prior IMPC vision studies employed qualitative, categorical assessments to identify genes associated with eye development using embryonic data [15] or with abnormal ocular morphology using adult eye dysmorphology data [16,17]. In many instances, these studies have connected the gene product to the eye for the first time. To date, ocular examination by the IMPC has been restricted to anatomical techniques that could readily be performed in a standardized fashion across the participating institutions. However, multiple ocular disease conditions such as achromatopsia [18], or congenital stationary night blindness [19] are characterized by functional loss without morphological alterations. Electroretinography (ERG) measures the electrical response of the retina to light stimuli and provides a noninvasive means to examine the functional properties of major outer retinal cell types [20,21]. Here we expand the standard IMPC phenotyping pipeline through the addition of ERG, screen 530 single-gene knockout mouse strains using that expanded pipeline, and report 30 strains that exhibited distinct and reproducible abnormalities involving outer retinal function.

## Results

### Increasing the impact of the IMPC on vision and ocular disease

To create a comprehensive catalogue of mammalian gene function, the IMPC has established a large-scale and high-throughput program to produce and phenotype single-gene targeted knockout mouse strains for every protein-coding gene in the mouse genome. In this study, we characterized 530 knockout strains (S1 Table) using the comprehensive IMPC primary phenotyping pipeline (https://www.mousephenotype.org/impress/index), which was supplemented at week 15 with the inclusion of ERG, an objective screen designed to identify functional ocular abnormalities (S1 Fig).

### Identifying genetic determinants of electrical signaling in the retina

Following overnight dark adaptation, mice at 106 ± 2 days of age [mean ± standard deviation (SD)] were examined using a standard ERG protocol (S2 Fig) comprised of two stimulus conditions. Under dark-adapted, scotopic conditions, the ERG waveform includes an initial cornea-negative a-wave generated by rod photoreceptors [22], followed sequentially by a positive polarity b-wave generated by rod depolarizing bipolar cells [23], the positive polarity c-wave reflecting the sum of a positive component generated by the apical RPE [24] and a typically smaller negative component generated by Müller cells [25], and finally a negative wave related to the fast oscillation (FO) representing a hyperpolarization of the basal RPE. The FO is typically analyzed with respect to a longer duration stimulus [24] and we refer to it here as ‘FO-like’. Under light-adapted, photopic conditions, the mouse ERG waveform includes an initial negative polarity a-wave reflecting activity of cone photoreceptors and inner retinal cells, followed by a positive b-wave reflecting the response of cone depolarizing bipolar cells [26]. The amplitude of these components was measured as described in S3 Fig. While latency data were collected and are available via the raw data repository on Zenodo (https://zenodo.org/records/16944276), our analysis focused on amplitude data. In addition to the 530 knockout strains examined, contemporaneous age and genetic background matched C57BL/6NJ inbred mice were tested as wildtype controls. In total, 4,723 individual mice underwent ERG testing. Given the size of this data set, a rigorous, multi-step data quality control (QC) workflow was employed (Methods; S4 Fig). Based on the predominant absence of sexual dimorphism in the wildtype control population, except for a moderate effect restricted to the right eye for the scotopic c- and FO-like wave (S5 Fig), we combined the data for each strain by sex. This increased the sample size and concomitantly the power to detect differences within each strain. A sample size threshold of n ≥ 5 mice per strain was established for inclusion in our downstream statistical analysis.

Upon conclusion of the QC process and application of the sample size threshold, ERG data were retained from 3,578 mutant mice across 530 knockout strains, along with 650 contemporaneous wildtype control mice. The results of statistical comparisons for all 530 knockout strains are presented in S1 Table and the Zenodo repository (https://zenodo.org/records/16944276).

**Fig 1:**
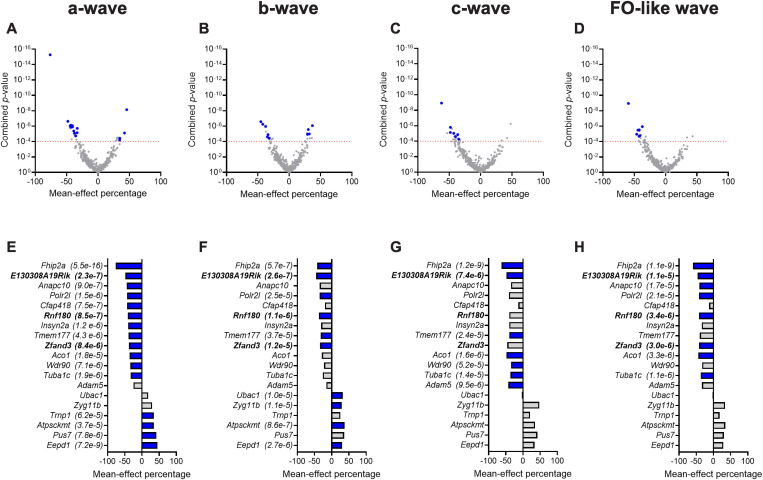
Summary of scotopic ERG data analysis. **(a-d)** Volcano plots for the four scotopic amplitude parameters: a-wave, b-wave, c-wave, and FO-like wave. Each panel plots the mean-effect percentage against the probability value determined for the four scotopic amplitude parameters (combined for both eyes) for each of 519 knockout strains tested. The *p*-value threshold profile required to identify a concordant, statistically significant strain was *p* ≤ 0.01 for each eye and *p* ≤ 0.0001 for data combined between the two eyes. The red horizontal dashed line indicates **p* *= 0.0001; blue symbols indicate concordant hits (i.e., detected in both eyes). Grey symbols represent genes that did not meet the criteria for statistical significance. In some cases, these genes had a combined p-value ≤ 0.0001 but were not classified as hits because the ERG phenotype was not concordant between eyes (i.e., p > 0.01 in one eye) and were therefore considered non-significant. **(e-h)** Waterfall plots listing the gene symbol from the 19 knockout strains for which a significant difference from wildtype was detected for any scotopic parameter. Blue indicates that the hit was significant for that parameter, and the associated combined *p*-value is provided in parenthesis after the gene symbol. Genes that were also significant for a photopic ERG parameter are indicated in bold.

Fig 1a-1d present volcano plots for the 519 single-gene knockout mouse strains that passed QC for the dark-adapted, scotopic ERG response and highlight the subset of 19 strains (represented by blue dots) that achieved statistical significance for at least one of the four scotopic amplitude parameters measured. Fig 1e-1h identify these strains, plotting the mean-effect percentage for each of the four waveform components assessed, and listing the combined *p*-value. We identified 16 strains with a significant change in a-wave amplitude (Fig 1e), 10 with changes in b-wave amplitude (Fig 1f), seven with changes in c-wave amplitude (Fig 1g), and eight with changes in FO-like wave amplitude (Fig 1h). Most of these scotopic hits represented decreased amplitude (negative mean-effect percentage), but a subset demonstrated increased amplitude. For example, 12 of the 16 scotopic hits involving the a-wave had a reduced amplitude response, while the remaining four had an increased a-wave amplitude.

**Fig 2:**
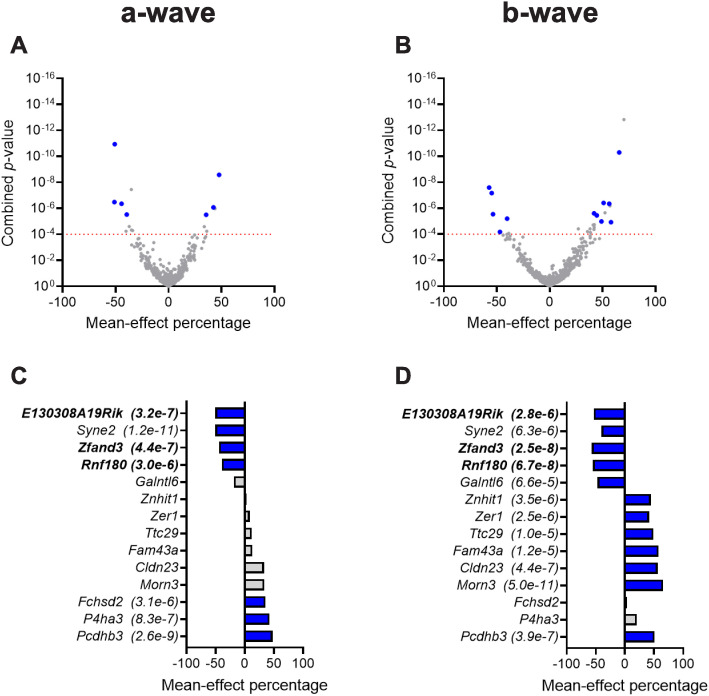
Summary of photopic ERG data analysis. **(a-b)** Volcano plots for the two photopic amplitude parameters: a-wave and b-wave. Both panels plot the mean-effect percentage against the probability value determined for the two photopic amplitude parameters (combined for both eyes) for each of 511 knockout strains tested. The *p*-value threshold profile required to identify a strain as statistically significant was *p* ≤ 0.01 for each eye and *p* ≤ 0.0001 for data combined between the two eyes. The red horizontal dashed line indicates **p* *= 0.0001; blue symbols indicate concordant hits (i.e., detected in both eyes). Grey symbols represent genes that did not meet the criteria for statistical significance. In some cases, these genes had a combined p-value ≤ 0.0001 but were not classified as hits because the ERG phenotype was not concordant between eyes (i.e., p > 0.01 in one eye) and were therefore considered non-significant. **(c-d)** Waterfall plots listing the gene symbol from the 14 knockout strains for which a significant difference from wildtype was detected for either photopic parameter. Blue indicates that the hit was significant for that parameter, and the associated combined *p*-value is provided in parenthesis after the gene symbol. Genes that were also significant for a scotopic ERG parameter are indicated in bold.

Fig 2a and 2b present volcano plots for the 511 single-gene knockout mouse strains that passed QC for the light-adapted, photopic ERG response and highlight the 14 strains (represented by blue dots) that achieved statistical significance in photopic a-wave and/or b-wave amplitude. Fig 2c and 2d identify these strains, plotting the mean-effect percentage for both components of the photopic ERG response and listing the combined *p*-value. We identified seven strains with a significant change in a-wave amplitude (Fig 2c), and 12 with changes in b-wave amplitude (Fig 2d). Five strains showed a decreased amplitude in their photopic response, the remaining nine strains showed an increased amplitude.

Three homozygous knockout mouse strains, C57BL/6NJ-*E130308A19Rik*^*em1(IMPC)J*^/Mmjax, C57BL/6NJ-*Rnf180*^*em1(IMPC)J*^/Mmjax, and C57BL/6NJ-*Zfand3*^*em1(IMPC)J*^/Mmjax, (highlighted in bold in Figs 1e-1h, 2c, and 2d) presented with significant reductions in both scotopic and photopic amplitudes. No strain was identified with a significant increase in both scotopic and photopic ERG response amplitude. In total, 30 unique strains presented with a statistically significant abnormality in at least one ERG parameter tested (Figs 1 and 2 and S1 Table). ERG waveforms for all mice tested for these 30 strains are presented in S6 Fig.

### Assessing sexual dimorphism in a subset of strains

Given the prevalence of sexual dimorphism [27], the typical IMPC study design allows for the evaluation of sex*genotype interactions, specifically whether one sex was more impacted by a given mutation than the other. In the current dataset, a subset of strains had a sufficient sample size (n ≥ 4 for each sex per strain) to test for sex*genotype interactions. Scotopic amplitude data from 124 strains (eight measures, four per eye) and photopic amplitude data from 117 strains (four measures, two per eye) were analyzed. A significance level of *p* ≤ 0.01 was used for each measure tested and the concordance requirement (combined left eye*right eye *p* ≤ 0.0001) was used to determine a significant hit. The analysis summarized in S2 Table showed that of the 496 scotopic measures tested (124 strains with four concordant measures), only one strain showed a significant sex*genotype interaction at one measure (c-wave amplitude for homozygous C57BL/6NJ-*Rcor3*^*em1(IMPC)J*^/Mmjax) but that this strain did not have a significant effect of genotype alone. None of the 234 photopic measures (117 strains with two concordant measures) showed significant, concordant interactions for sex*genotype. The absence of clear evidence of sexual dimorphism within this subset of mutant strains reinforced our decision to combine data across sexes to increase statistical power.

### Internal controls within gene set

The 530 genes chosen for knockout allele generation and characterization through the IMPC pipeline were selected based upon broad criteria. These typically included genetically tractable genes with human orthologues and those for which no existing knockout mouse strain was freely available to the scientific community. No phenotype- or disease-specific criteria were included that would actively bias these 530 genes with respect to eye disease. Of the 30 genes with statistically significant abnormal ERG results, two (*Cfap418* and *Syne2*; Figs 1 and 2 and S1 Table) had been previously reported, validating the ability of our ERG screen to detect true retinal defects.

**Fig 3:**
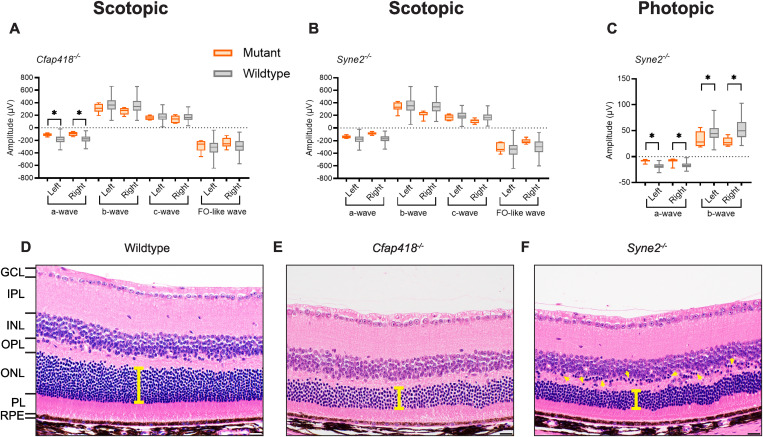
Known genetic determinants of retinal function. Two genes, *Cfap418* and *Syne2*, known to impact retinal function, were detected among the 530 knockout strains tested and served as internal controls. **(a-c)** Mean shown inside quartile boxplots (error bars indicate minimum and maximum values) for homozygous knockout (orange) C57BL/6NJ-*Cfap418*^*em1(IMPC)J*^/Mmjax (n = 6) **(a)**, and C57BL/6NJ-*Syne2*^*em1(IMPC)J*^/Mmjax (n = 7) (b-c) mice, and their associated windowed, wildtype controls (grey) for each ERG response amplitude parameter tested. Only wildtype mice with a window weight of 1.0 (rounded to three significant figures) were included in these plots. This selection resulted in the following wildtype group sizes: panel a, by waveform order (a-, b-, c-, and FO-like wave) for left (n = 428, 412, 219, and 148) and right (n = 231, 231, 116, and 231) eye; panel b, by waveform order (a-, b-, c-, and FO-like wave) for left (n = 588, 600, 203, and 191) and right (n = 554, 554, 306, and 306) eye; panel c, by waveform order (a- and b-wave) for left (n = 193 and 57) and right (n = 171 and 97) eye. Left and right eye data are shown separately. Insufficient photopic data were available for strain C57BL/6NJ-*Cfap418*^*em1(IMPC)J*^/Mmjax to trigger statistical analysis. * indicates a concordant significant difference between mutant and wildtype (combined *p* ≤ 0.0001). **(d-f)** Representative H&E-stained axial sections adjacent to the optic nerve head of right (OD) eyes from wildtype (d, n = 11 mice, 22 eyes), C57BL/6NJ-*Cfap418*^*em1(IMPC)J*^/Mmjax homozygous knockout (e, n = 4 mice, 8 eyes), and C57BL/6NJ-*Syne2*^*em1(IMPC)J*^/Mmjax homozygous knockout (f, n = 6 mice, 12 eyes) mice. GCL: Ganglion cell layer, IPL: inner plexiform layer, INL: inner nuclear layer, OPL: outer plexiform layer, ONL: outer nuclear layer, PL: photoreceptor layer, RPE: retinal pigment epithelium. The scale bar represents 20 µm. The yellow bars highlight the difference in ONL thickness, while yellow arrowheads in panel (f) highlight mislocalized photoreceptor nuclei.

*C8orf37*, the human orthologue of *Cfap418* (cilia and flagella associated protein 418), is associated with several autosomal recessive human conditions including cone-rod dystrophy, retinitis pigmentosa with early macular involvement, and Bardet-Biedl syndrome (https://web.sph.uth.edu/RetNet/) [28–31]. Homozygous C57BL/6NJ-*Cfap418*^*em1(IMPC)J*^/Mmjax mice displayed a comparable phenotype with a significant reduction in scotopic a-wave amplitude (Fig 3a; mean-effect percentage of -43.2%; **p* *= 7.5 × 10^-7^). While also reduced, the remaining scotopic parameters did not achieve statistical significance. Following our robust QC process, insufficient photopic data were available from the C57BL/6NJ-*Cfap418*^*em1(IMPC)J*^/Mmjax strain to trigger statistical analysis. ERG waveforms for the C57BL/6NJ-*Cfap418*^*em1(IMPC)J*^/Mmjax strain are presented in S6 Fig. Complementing the ERG data, retinal histopathology of the left and right eyes from four mice (eight eyes) revealed diffusely decreased retinal thickness (Fig 3e), compared to wildtype mice (n = 11 mice, 22 eyes; Fig 3d). Specifically, we observed decreased photoreceptor cell nuclei in the outer nuclear layer (ONL), and a reduction in the thickness of the outer plexiform (OPL) and photoreceptor (PL) layers (Fig 3e). These changes were consistent in severity between left and right eyes and across all *Cfap418*^*em1(IMPC)J*^/Mmjax homozygous mutant mice, with no apparent differences between sexes. To quantify these histological observations, we counted the mean number of photoreceptor nuclei within 25µm-wide ONL segments located 0.5 mm from the center of the optic nerve head, on both sides of the optic nerve. We found a statistically significant reduction in photoreceptor nuclei in homozygous mutant mice (C57BL/6NJ-*Cfap418*^*em1(IMPC)J*^/Mmjax; n = 4 mice, 7 eyes; mean ± SD = 39.4 ± 3.9 nuclei) compared to wildtype controls (C57BL/6NJ; n = 11 mice, 21 eyes; mean ± SD = 68.9 ± 6.7 nuclei; *p* = 2.5 × 10^-14^; S7 Fig). The decreases in outer retinal thickness and photoreceptor nuclei were consistent with the decreased amplitude of scotopic ERG responses.

A previous photopic ERG screen identified a spontaneous mutant involving a premature stop codon in *Syne2* (spectrin repeat containing, nuclear envelope 2) [32]. Consistent with that model, C57BL/6NJ-*Syne2*^*em1(IMPC)J*^/Mmjax homozygous mice displayed a statistically significant reduction in the amplitude of the photopic a-wave (mean-effect percentage of -50.8%; *p* = 1.2 × 10^-11^) and the photopic b-wave (mean-effect percentage of -40.3%; *p* = 6.3 × 10^-6^) (Fig 3c). Scotopic responses were not significantly different from wildtype (Fig 3b), in line with the prior report of a greater impact on the photopic than the scotopic ERG [32]. ERG waveforms for the C57BL/6NJ-*Syne2*^*em1(IMPC)J*^/Mmjax strain are presented in S6 Fig. Retinal histopathology of the left and right eyes from six mice (12 eyes) revealed ONL thinning and photoreceptor cell mislocalization in the OPL (Fig 3f), when compared to wildtype mice (n = 11 mice, 21 eyes; Fig 3d). These changes were consistent in severity between left and right eyes and across all *Syne2*^*em1(IMPC)J*^/Mmjax homozygous mutant mice, with no apparent differences between sexes. We performed the same quantitative analysis of photoreceptor nuclei as described above for homozygous mutant mice (C57BL/6NJ-*Syne2*^*em1(IMPC)J*^/Mmjax, *n* = 6 mice, 11 eyes) and observed a significant reduction in photoreceptor nuclei compared to wildtype controls (mutant mean ± SD = 49.2 ± 4.4 nuclei; *p* = 2.6 × 10^-10^; S7 Fig).

### Novel genetic determinants of retinal dysfunction

Of the 30 hits in which statistically significant ERG abnormalities were identified, 28 represent novel findings that link the targeted disruption of these genes to altered retinal function for the first time. Two examples are detailed herein.

**Fig 4:**
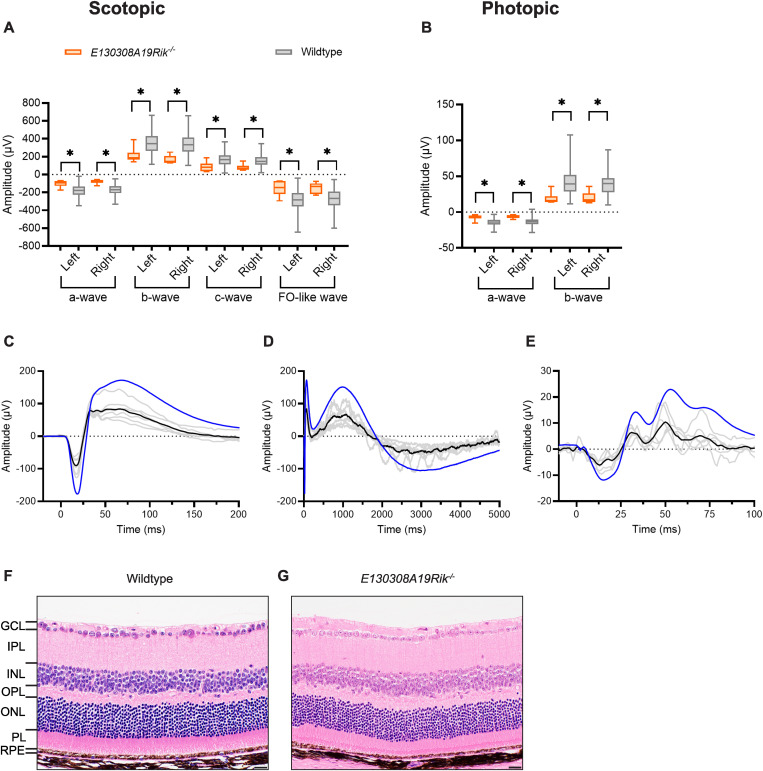
*E130308A19Rik*, a novel genetic determinant of retinal function. Abnormalities in scotopic and photopic ERG responses were observed following inactivation of gene *E130308A19Rik*. **(a-b)** Mean shown inside quartile boxplots (error bars indicate minimum and maximum values) for homozygous knockout (orange) C57BL/6NJ-*E130308A19Rik*^*em1(IMPC)J*^/Mmjax mice (n = 6) and their windowed, wildtype controls (grey), showing ERG response amplitude parameters for left and right eyes separately. Only wildtype mice with a window weight of 1.0 (rounded to three significant figures) were included in these plots. This selection resulted in the following wildtype group sizes: panel a, by waveform order (a-, b-, c-, and FO-like wave) for left (n = 420, 524, 397, and 337) and right (n = 420, 420, 397, and 552) eye; panel b, by waveform order (a- and b-wave) for left (n = 194 and 371) and right (n = 194 and 185) eye. * indicates concordant significant differences between mutant and wildtype (combined *p* ≤ 0.0001). **(c-e)** Average traces (left and right eye combined) for individual mutant mice (solid grey) and the mutant strain average (solid black) compared to averages from all QC-passed wildtype mice (solid blue; scotopic n = 631, photopic n = 624). Scotopic waveforms are shown in two timescales: 225 ms (-25 to 200 ms) (c) highlighting a-wave and b-waves, and 5000 ms (0 to 5000 ms) (d) to visualize the c- and FO-like waves. Photopic waveforms (e) shown as 112.5 ms (-12.5 to 100 ms) of response highlighting a- and b-waves. **(f-g)** Representative H&E-stained axial sections adjacent to the optic nerve head of OD eyes from C57BL/6NJ-*E130308A19Rik*^*em1(IMPC)J*^/Mmjax homozygous knockout (g, n = 4 mice, 8 eyes), and wildtype (f, n = 11 mice, 22 eyes) mice. GCL: Ganglion cell layer, IPL: inner plexiform layer, INL: inner nuclear layer, OPL: outer plexiform layer, ONL: outer nuclear layer, PL: photoreceptor layer, RPE: retinal pigment epithelium. The scale bar represents 20 µm.

Mice homozygous for inactivation of RIKEN cDNA E130308A19 gene (C57BL/6NJ-*E130308A19Rik*^*em1(IMPC)J*^/Mmjax) presented with a grossly abnormal ERG response. To emphasize the concordant nature of the abnormalities, boxplots of ERG response amplitude measurements are presented separately for the left and right eyes for each of the four scotopic parameters (Fig 4a) and the two photopic parameters (Fig 4b). ERG response amplitude was significantly reduced in both eyes for all parameters. To further highlight the ERG abnormalities, waveforms are presented (Figs 4c-4e and S6). Scotopic waveforms are shown on different timescales to emphasize the reduction in amplitude relative to wildtype control mice of the earlier a- and b-waves (Fig 4c) and the later c- and FO-like waves (Fig 4d). The photopic waveform shows a clear overall reduction in amplitude compared to wildtype control mice (Fig 4e). ERG analysis was performed on data from only three male and three female C57BL/6NJ-*E130308A19Rik*^*em1(IMPC)J*^/Mmjax homozygous mutant animals, therefore sexual dimorphism of the results could not be assessed. A feature of the C57BL/6NJ-*E130308A19Rik*^*em1(IMPC)J*^/Mmjax strain was that the functional defects identified by ERG were not accompanied by a marked structural phenotype. Histologically, the retinas of C57BL/6NJ-*E130308A19Rik*^*em1(IMPC)J*^/Mmjax homozygous mutant mice (n = 4 mice, 8 eyes; Fig 4g) were similar to those of wildtype control mice (n = 11 mice, 22 eyes; Fig 4f). Furthermore, homozygous mutant mice (C57BL/6NJ-*E130308A19Rik*^*em1(IMPC)J*^/Mmjax, n = 4 mice, 6 eyes) did not show a significant reduction in photoreceptor nuclei compared to wildtype controls (mutant mean ± SD = 71.5 ± 5.1 nuclei; *p* = 1.0; S7 Fig). The only other abnormality identified from the full IMPC early adult pipeline was a sexually dimorphic increase in plasma total cholesterol and high-density lipoprotein cholesterol, observed in *E130308A19Rik*^*em1(IMPC)J*^/Mmjax homozygous males but not females (https://www.mousephenotype.org/data/genes/MGI:2442164). A knockout mutation for the gene *E130308A19Rik* has been previously reported (https://en.gempharmatech.com/product/details100035_4030910.html) but phenotypic characterization was not included. The human orthologue, *KIAA1958*, has no strong link to human disease and no link to outer retinal disease (https://diseases.jensenlab.org, https://retnet.org).

The lack of a structural phenotype correlating with the detected physiological abnormality was more common than anticipated. Histopathology review was performed on eyes from at least one male mouse (two eyes) up to a maximum of eight mice (16 eyes), aged 17–19 weeks, for all 30 knockout mouse strains presenting with abnormal ERG, and compared with wildtype control mice (n = 11 mice, 22 eyes). Besides the two internal control strains reported in Fig 3, only one additional strain, knocked out for the *Fhip2a* gene (described below), presented with abnormal retinal histopathology. Broader assessment of the histopathology of the whole eye, including anterior segment (adnexa, cornea, iris, and lens) and posterior segment (vitreous, choroid, sclera, and optic nerve), indicated no significant strain-level abnormalities beyond those already mentioned for the retina. Of the 30 knockout strains with statistically significant ERG abnormalities, 17 strains fulfilled the inclusion criteria (correct optic nerve head orientation in n ≥ 3 mice) to perform quantitative analysis of the photoreceptor nuclei counts. Aligning with manual histopathology review, this quantitative analysis identified a statistically significant reduction in photoreceptor nuclei count only for knockouts of *Cfap418*, *Syne2*, and *Fhip2a* (S7 Fig).

**Fig 5:**
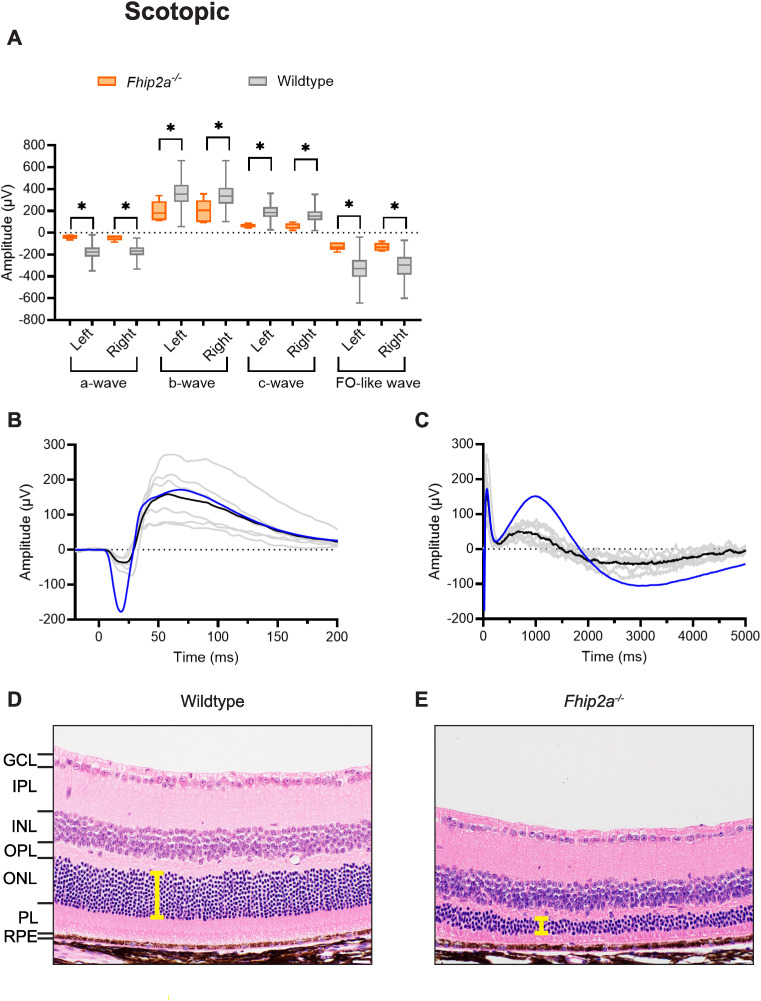
*Fhip2a*, a novel genetic determinant of retinal function. Abnormalities in scotopic ERG responses were observed following inactivation of gene *Fhip2a*. **(a)** Mean shown inside quartile boxplots (error bars indicate minimum and maximum values) for homozygous knockout (orange) C57BL/6NJ-*Fhip2a*^*em1(IMPC)J*^/Mmjax mice (n = 6) and their windowed, wildtype controls (grey), showing ERG response amplitude parameters for left and right eyes separately. Only wildtype mice with a window weight of 1.0 (rounded to three significant figures) were included in these plots. This selection resulted in the following wildtype group sizes in panel a, by waveform order (a-, b-, c-, and FO-like wave) for left (n = 588, 588, 243, and 254) and right (n = 554, 554, 554, and 254) eye. * indicates concordant significant differences between mutant and wildtype (combined *p* ≤ 0.0001). Insufficient photopic data were available for strain C57BL/6NJ-*Fhip2a*^*em1(IMPC)J*^/Mmjax to trigger statistical analysis. **(b-c)** Average traces (left and right eye combined) for individual mutant mice (solid grey) and the mutant strain average (solid black) compared to averages from all QC-passed wildtype mice (solid blue; scotopic n = 631). Scotopic waveforms are shown in two timescales: 225 ms (-25 to 200 ms) (b) highlighting a-wave and b-waves, and 5000 ms (0 to 5000 ms) (c) to visualize the c- and FO-like waves. **(d-e)** Representative H&E-stained axial sections adjacent to the optic nerve head of OD eyes from C57BL/6NJ-*Fhip2a*^*em1(IMPC)J*^/Mmjax homozygous knockout (e, n = 8 mice, 15 eyes), and wildtype (d, n = 11 mice, 22 eyes) mice. GCL: Ganglion cell layer, IPL: inner plexiform layer, INL: inner nuclear layer, OPL: outer plexiform layer, ONL: outer nuclear layer, PL: photoreceptor layer, RPE: retinal pigment epithelium. The scale bar represents 20 µm. The yellow bars highlight the difference in ONL thickness.

Mice homozygous for inactivation of FHF complex subunit HOOK interacting protein 2A (C57BL/6NJ-*Fhip2a*^*em1(IMPC)J*^/Mmjax) presented with a statistically significant reduction in the amplitude of all scotopic parameters. To emphasize the concordant nature of the abnormalities, boxplots of ERG response amplitude measurements are presented separately for the left and right eyes for each of the four scotopic parameters (Fig 5a). To further highlight the ERG abnormalities, scotopic waveforms are shown (Figs 5b-5c and S6) on different timescales to emphasize the reduction in amplitude relative to wildtype control mice of the earlier a- and b-waves (Fig 5b) and the later c- and FO-like waves (Fig 5c). Note that while the averaged mutant b-wave intersects closely with the wildtype control (Fig 5b), the b-wave amplitude is calculated from the trough of the a-wave to the peak of the b-wave (S3 Fig), and therefore the measured amplitude is significantly reduced. Insufficient photopic data passed our QC process to conduct a statistical analysis. Retinal histopathology of the left and right eyes of eight mice (15 eyes, the right eye from one animal was not available) revealed diffusely decreased retinal thickness, including decreased photoreceptor cell nuclei in the ONL, decreased thickness of the OPL, fusion of the inner and outer nuclear layers, and thinning of the photoreceptor layer (Fig 5e) compared to wildtype mice (n = 11 mice, 22 eyes; Fig 5d). These changes were consistent in severity between left and right eyes and across all C57BL/6NJ-*Fhip2a*^*em1(IMPC)J*^/Mmjax homozygous mutant mice, with no apparent differences between sexes. We performed the same quantitative analysis of photoreceptor nuclei as described above for homozygous mutant mice (C57BL/6NJ-*Fhip2a*^*em1(IMPC)J*^/Mmjax, *n* = 7 mice, 14 eyes) and observed a significant reduction in photoreceptor nuclei count compared to wildtype controls (mutant mean ± SD = 18.4 ± 2.2 nuclei; *p* = 9.9 × 10^-15^; S7 Fig). Phenotyping results for mice homozygous for the *Fhip2a*^*em1(IMPC)J*^ allele assessed through the full IMPC early adult pipeline are available (https://www.mousephenotype.org/data/genes/MGI:2147545). Abnormalities include two qualitative eye phenotypes: persistence of the hyaloid vascular system (present in 6/6 males but 0/8 females assessed) and abnormal optic disc morphology (3/8 females were abnormal while 6/6 males were normal).

No other *Fhip2a* mutant mouse strain has been reported. While no human diseases have been directly associated with *FHIP2A* (synonym *FAM160B1*), a likely pathogenic link to Syndromic Intellectual Disability has been reported [33].

### Assessing the specificity of the ERG screen

To determine if our ERG screen overlooked any known human or murine genes important for outer retina function, we cross-referenced the list of 530 genes reported here with two curated databases.

We first compared our gene list to the Retinal Information Network (RetNet Database, queried January 17, 2025; https://retnet.org/) list of 346 gene and locus symbols associated with inherited retinal diseases in humans. Ten overlapping genes were found (*Ccdc51*, *Cep78*, *Cfap418*, *Dhx38*, *Ensa*, *Exosc2*, *Nphp3*, *Tbc1d32*, *Zfp423*, *Zfp513*). Of these, *Cfap418* was the only one identified in the current study as influencing ERG response amplitude.

Next, we queried MouseMine (https://www.mousemine.org; May 14, 2024) for genes annotated with two key parental Mammalian Phenotype (MP) ontology terms and all their associated sibling terms in the MP hierarchy: ‘abnormal eye electrophysiology’ (MP:0005551) and ‘abnormal electroretinogram waveform feature’ (MP:0012029). Of the 349 unique gene symbols associated with these terms, four were included in our screen: *Atp8a2*, *Cep78*, *Cfap418*, and *Syne2*. Two of these (*Cfap418* and *Syne2*) were identified as ERG hits in our study.

### Identifying a genetic model for an inherited retinal disease of unknown etiology

**Fig 6:**
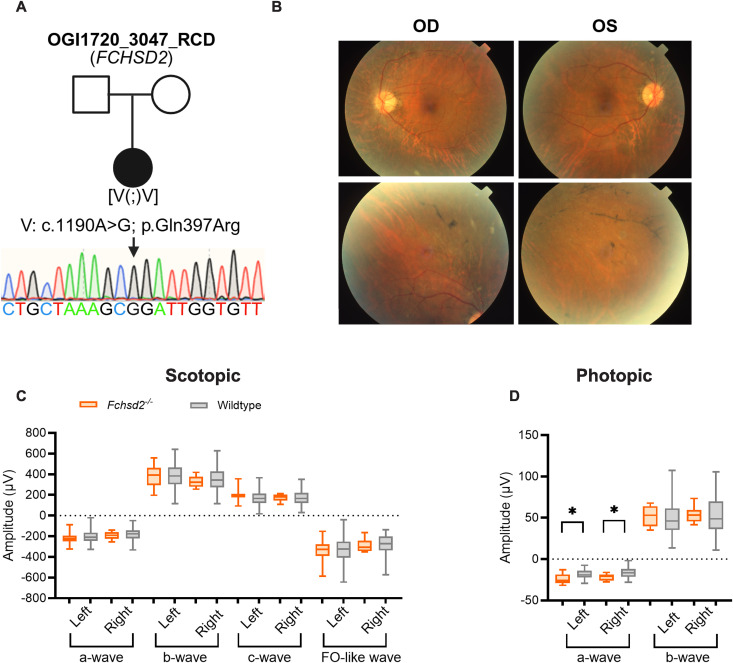
*FCHSD2* human variant. **(a)** Ophthalmic Genomics Institute (OGI) pedigree of a patient carrying a homozygous variant in *FCHSD2*. The female proband is represented with a black circle and the clinical diagnosis is mentioned above the pedigree (RCD, rod-cone degeneration). The **c.**1190A > G variant (V1) was confirmed by Sanger sequencing (arrow), although segregation could not be performed due to the lack of parental DNA, thus the variant is indicated as [V1(;)V1]. **(b)** Fundus photographs show attenuated retinal vessels and sparse bone spicule pigmentation in the periphery of both eyes. OD: right eye, OS: left eye. Scotopic (n = 10) and photopic (n = 9) ERG responses were measured in *Fchsd2* knockout mice. **(c-d)** Mean shown inside quartile boxplots (error bars indicate minimum and maximum values) for homozygous knockout (orange) C57BL/6NJ-*Fchsd2*^*em1(IMPC)J*^/Mmjax mice and their windowed, wildtype controls (grey), showing ERG response amplitude parameters for left and right eyes separately. Only wildtype mice with a window weight of 1.0 (rounded to three significant figures) were included in these plots. This selection resulted in the following wildtype group sizes: panel c, by waveform order (a-, b-, c-, and FO-like wave) for left (n = 109, 212, 108, and 153) and right (n = 212, 212, 274, and 69) eye; panel d, by waveform order (a- and b-wave) for left (n = 102 and 125) and right (n = 53 and 102) eye. * indicates concordant significant differences between mutant and wildtype (combined **p* *≤ 0.0001).

Although enormous progress has been made in human retinal gene discovery, numerous individuals are still affected by inherited retinal diseases (IRDs) of unknown genetic etiology. To assess the clinical relevance of the novel mouse strains identified here with significant abnormalities in ERG response amplitude, we evaluated these as candidate genes in a cohort of approximately 800 IRD patients who underwent exome sequencing and/or genome sequencing. In doing so, we identified a female patient previously diagnosed with rod-cone degeneration (OGI1720_003047) who is homozygous for a missense variant in *FCHSD2* (c.1190A > G; p.Gln397Arg; Fig 6a); the IMPC knockout allele, C57BL/6NJ-*Fchsd2*^*em1(IMPC)J*^/Mmjax, is shown here to have an abnormal ERG component.

The p.Gln397Arg variant is rare, with a minor allele frequency of 0.00002087 in the GnomAD-v4 population database [34] (https://gnomad.broadinstitute.org), and it has never been found in homozygosity. Gln397 corresponds to the last amino acid residue of the coiled-coil structure located between the main domains of the FCHSD2 protein (the F-BAR domain at N-terminal and the SH3 domain at C-terminal) [35]. This residue is conserved across species, based on the 100 vertebrate sequences alignment available in the UCSC Genome Browser [36], and its change to arginine has an elevated Combined Annotation Dependent Depletion (CADD) score [37] of 26. It was also predicted to be deleterious by the pop-EVE [38] algorithm (score of -3.6, corresponding to the autosomal recessive mean pathogenic ClinVar [39] score predicted by this tool). Since this is the only case with a rare variant in that gene, it currently remains classified as a variant of unknown significance according to the American College of Medical Genetics guidelines [40].

The patient was clinically evaluated at the age of 32. She reported no family history of retinal disease. Her best-corrected visual acuity was mildly reduced (20/25 OD; 20/30 OS). Goldmann visual fields were full to the larger V4e target in both eyes but constricted to the smaller I4e target (70 degrees in the right eye and 20 degrees in the left eye). A dilated exam revealed attenuated retinal vessels and sparse bone spicule pigmentation in the periphery of both eyes (Fig 6b). Full-field ERG showed bilateral yet asymmetrical reduction of retinal function, which was more prominent in cones than rods.

Mice homozygous for inactivation of FCH and double SH3 domains 2 (C57BL/6NJ-*Fchsd2*^*em1(IMPC)J*^/Mmjax) presented with no change in the amplitude of any dark-adapted ERG components (Fig 6c). Under photopic conditions, the a-wave was significantly increased in amplitude (Fig 6d). S6 Fig displays the scotopic and photopic waveforms for this strain. The eye histopathology for these mice was unremarkable and no significant change in photoreceptor nuclei count was noted (S7b Fig), suggesting no structural abnormalities at 17–19 weeks of age.

## Discussion

We performed a large-scale ERG-focused analysis within the context of the IMPC phenotyping pipeline. The goal of the IMPC is to identify genes that play critical roles in key biological systems, and multiple systems have been examined [41–43] including the eye [15–17]. Considering important conditions that affect outer retinal function but either largely (cone dystrophy) or completely (congenital stationary night blindness) spare the overall retinal structure, we added an ERG-based functional screen to the standard IMPC pipeline. This screen is sensitive to these and related conditions and provides critical information for clinical diagnosis [20]. Out of 530 single-gene knockout mouse strains examined, representing 2.5% of all protein-coding genes, we identified 30 (5.7%) with an ERG abnormality. Among the identified hits were two genes, *Cfap418* and *Syne2*, which are known to play a role in the retina [28–32]. This finding validates the effectiveness of our screening method in detecting true retinal defects.

Although 10 genes overlapping with RetNet and/or annotated with relevant MP terms were not identified as a hit in our screen (*Atp8a2*, *Ccdc51*, *Cep78*, *Dhx38*, *Ensa*, *Exosc2*, *Nphp3*, *Tbc1d32*, *Zfp423*, *Zfp513*), several of these may have been missed due to study design constraints. Five of the genes we examined have been linked to recessive eye diseases in humans but were tested in this study as heterozygotes due to homozygous embryonic lethality (*Dhx38*, *Exosc2*, *Tbc1d32*, *Zfp423*) or severe preweaning lethality (*Nphp3*). A frameshift mutation predicted to produce a nonfunctional CCDC51 protein has been reported to cause rod-cone dysfunction in humans [44]. The authors noted that this dysfunction is mild enough that it may not be replicated in mouse models. ATP8A2 is localized to the photoreceptor outer segments, and homozygous null *Atp8a2* mice have been reported with shortened outer segments, photoreceptor degeneration, and ERG amplitude reductions [45]. In the same study, the ERG responses of heterozygous mice were indistinguishable from those of the wildtype control animals [45]. The *Atp8a2* heterozygotes studied here due to the shortened lifespan of homozygous knockout mice showed normal ERG responses, consistent with Coleman *et al*. [45]. The remaining three overlapping genes appear to be true ‘misses’ and include *Cep78* and *Ensa*, which have been previously associated with age-dependent, progressive photoreceptor degeneration in knockout mice [46–48] and *Zfp513*, which has not been previously linked to ocular disease in the mouse but is associated with autosomal recessive retinitis pigmentosa in humans and loss of photoreceptors in a zebrafish knockdown model [49].

Factors like allele design, genetic background, age at testing, ERG test protocol, or screen sensitivity could explain these misses. Our analysis of ‘misses’ compared to the RetNet and MP ontology databases suggests that we may be conservative in classifying an abnormal ERG response as a hit, which is appropriate for a large-scale effort.

In comparing the data obtained from knockout strains to wildtype controls, we identified two classes of abnormalities: increased and decreased ERG response amplitudes. The predominant pattern of abnormality in both classes originated in the photoreceptor-driven a-wave and then cascaded to the later ERG components, which depend on photoreceptor function. No knockout strain exhibited a selective reduction in the scotopic b-wave, which would implicate an important role for the gene in depolarizing bipolar cells themselves, the photoreceptor-to-bipolar cell synapse, or in vascular support of the outer retina, as seen in multiple mouse models [50]. Each of the models that exhibited a significant decrease in the c-wave and/or FO-like wave also had a decreased scotopic a-wave, indicating that these reductions were likely secondary to the decreased response of rod photoreceptors, which is required for generation of the c-wave and FO in mice [51].

While three of the models with amplitude reduction had a corresponding anatomical abnormality in the retina detected by histopathology assessment, the remaining 12 did not. Although histopathology evaluation has been recognized as instrumental in identifying unique phenotypes in high throughput mouse phenotyping screens [52,53], it focuses on anatomic changes and may not detect changes in physiological processes important for retinal function, such as phototransduction, ciliary trafficking, neurotransmission, visual retinal cycle, some lipid metabolism, extracellular matrix turnover, transepithelial transport, oxidative stress processes, and general signaling processes [54]. It is also important to note that histopathology was performed at 17–19 weeks of age, a relatively early time point at which functional abnormalities may precede anatomic changes. Many retinal degenerative phenotypes emerge later, and histological correlates might have been detected had old mice been assessed in the current study. Additionally, while H&E staining facilitated the identification of microscopic structural defects, more sensitive eye imaging methods may be required to detect subtler changes. These considerations highlight the importance of utilizing both functional (ERG) and structural (histologic) retina evaluation in high throughput phenotyping screens, while also recognizing the influence of timing and methodological sensitivity on phenotype detection.

Multiple knockout strains had increased ERG response amplitudes. In the literature, most ERG studies of mouse models have focused on genes that are expected to induce a photoreceptor degeneration phenotype or compromise photoreceptor function. While an increase in ERG response amplitude is rare, it is not unknown [55–57]. This highlights the biological value of an unbiased, hypothesis-generating screen to uncover the relative prevalence of a largely uninvestigated body of genes that cause an increase in ERG response amplitude. The mechanisms driving this biological outcome remain to be determined, however, the increase in ERG response amplitudes were not associated with an increase in the number of photoreceptors. To our knowledge, none of the genes encode proteins that are known to be involved in phototransduction or its deactivation. Possible mechanisms may involve a role in establishing the resistance profile of the sclera or some other tissue that impacts ocular resistance, thereby affecting the amplitude of the ERG field potential.

The ERG screen used in this study was designed to efficiently examine the function of key outer retinal cell types. As a result, we did not evaluate the functional properties of all retinal cell types. Furthermore, while ERG protocols exist to probe important properties of the outer retina including dark adaptation via response recovery following a bleach [58,59], and deactivation of phototransduction using a paired flash protocol [60,61], these were not included in our screen. As a result, genes that impact these processes would be missed.

A notable feature of the present study is that it effectively serves as a sensitized screen. This is because the C57BL/6NJ background strain used by IMPC carries the *Crb1*^*rd8*^ mutation, which leads to progressive photoreceptor degeneration on this genetic background [62]. This was controlled by using genetic background-matched, C57BL/6NJ wildtype mice as the comparison group, and by assessing ERG when the mice were relatively young (approximately 15 weeks of age). Some of the hits reported here could be modifiers of the *Crb1*^*rd8*^ retinal degeneration phenotype. To assess this, it will be important to evaluate hits on a C57BL/6NJ-*Crb1*^*rd8+em1Mvw*^/MvwJ background, which does not exhibit *Crb1*^*rd8*^ retinal dysplasia [63,64].

We evaluated our hits as candidate genes for human disease. In a cohort of 800 patients confirmed to have a phenotype consistent with an inherited retinal disease, we identified a patient with retinal degeneration and a rare, homozygous missense variant in *FCHSD2*, the human orthologue of a hit identified here, *Fchsd2*. We encourage other groups with similar cohorts of patients without a molecular diagnosis to evaluate the altered ERG response amplitude hits reported here.

In conclusion, our study identified a total of 30 single-gene knockout mouse strains with significantly altered ERG response amplitudes. Notably, two of these protein-coding genes have previously been implicated in outer retinal dysfunction, validating our ERG screening protocol as an effective internal control. While beyond the scope of this study, future research should focus on elucidating the roles of the encoded proteins in cellular function, including the specific cells in which they are expressed, the temporal dynamics of their expression, and their cellular distribution. This set of genes, associated with altered ERG phenotypes in mice, holds significant potential to bridge the gap between genetic loci implicated in inherited retinal degeneration and those confirmed as human disease genes.

## Methods

### Ethics statement

All mouse work reported here was conducted at the Jackson Laboratory under the Institutional Animal Care and Use Committee-approved license numbers 14,004 and 11,005, AAALACi accreditation number 00096, and NIH Office of Laboratory Animal Welfare assurance number D16-00170. Genetic screening of patient samples was approved by the institutional review board at the Massachusetts Eye and Ear, an affiliate of Mass General Brigham (MGB) healthcare system (Human Studies Committee MGB, Boston, MA, USA) and complied with the Health Information Portability and Accessibility Act. All aspects of the project adhered to the tenets of the Declaration of Helsinki. Informed consent was obtained from all individuals on whom genetic testing and further molecular evaluations were performed.

### Animals

Mice carrying single-gene knockout alleles were generated via CRISPR/Cas9 mediated genome editing using standard techniques [65]. In total, data from 530 knockout mouse strains (listed in S1 Table) were included in this study. The gene selection process was based on broad criteria, including genetic tractability and the presence of human orthologues, without existing mouse knockout strains. No specific criteria related to phenotypes or diseases were applied, therefore there was no active bias towards eye disease. All knockout mouse strains were produced and maintained on a C57BL/6NJ genetic background (IMSR_JAX:005304, The Jackson Laboratory, Bar Harbor, ME, USA) which carries the *Crb1*^*rd8*^ mutation [62]. Mutant allele production details, including guide sequences, can be found at www.mousephenotype.org by searching for the gene symbol. Homozygous animals were tested when viable and available (370 knockout strains), including fourteen X-linked genes. For the remaining 160 knockout strains, heterozygous animals were tested. Strain zygosity is presented in S1 Table. For brevity, genotype is abbreviated in S1 Table and within figures as *Gene symbol*^*-/-*^ and *Gene symbol*^*+/-*^ for homozygosity and heterozygosity, respectively.

Mice were maintained in a specific pathogen-free barrier using a 12-hour light–dark schedule, and experiments were conducted in the light phase. Mice had *ad libitum* access to water and food (5K52 diet, LabDiet, Gray Summit, MO, USA). A housing density of 1–5 animals per cage was maintained in individually ventilated cages [Thoren Duplex II Mouse Cage #11 and Thoren Maxi-Miser PIV System (overall dimensions of caging: [L × W × H]: 308 × 308 × 162 mm)]. Wood shavings (aspen or pine) bedding substrate was provided and on rare occasions when mice were housed individually, their home cages were supplemented with a nestlet and cardboard hut. Room temperature and humidity were maintained between 20–22°C and 44–60%, respectively.

### International mouse phenotyping consortium

This study was conducted as part of the International Mouse Phenotyping Consortium [66], an initiative aimed at determining the function of the orthologous mammalian genome by generating and phenotypically characterizing single-gene knockout mouse strains. The adult phenotyping pipeline used by the IMPC, which includes multiple assays covering various biological domains such as behavior, cardiac function, metabolism, sensory (auditory and visual), and hematology, is outlined in S1 Fig.

### Mouse phenotyping data acquisition

Mutant and wildtype control mice were enrolled into adult phenotyping at four weeks of age. Each week, five male and five female C57BL/6NJ inbred mice (IMSR_JAX:005304, The Jackson Laboratory, Bar Harbor, ME, USA) were tested. These mice served as wildtype control animals for all mutant mice examined that week and were also used for the windowing control strategy [67] described below. For each knockout strain, eight male and eight female mutant mice were enrolled into the adult phenotyping pipeline.

*In vivo* eye dysmorphology phenotyping was conducted when mice were 15 weeks of age (S1 Fig) as described previously [16].

ERG was conducted at 15 weeks of age, and the protocol is summarized in S2 Fig. After overnight dark adaptation, mice were anesthetized with 2% isoflurane, and pupils were dilated with eyedrops (typically 0.2% Cyclomydril ophthalmic solution, internal Jackson Laboratory pharmacy). Mice were placed on the Celeris Rodent Platform heater, programmed to maintain the subject’s core body temperature at 37 °C ± 1 °C, and ERG responses were recorded using a Celeris system (Diagnosys LLC, Lowell MA, USA). The Celeris electrode-stimulators were wetted with methylcellulose (Goniovisc-Hypromellose 2.5%, internal Jackson Laboratory pharmacy) and placed in light contact with the corneal surface of each eye. ERG responses were recorded using an amplifier with a bandpass of 0.125 to 50 Hz and a sampling rate of 2,000 Hz. ERG responses were recorded from both eyes, with the unstimulated eye serving as a reference for the stimulated eye. For scotopic measurement, the flash stimulus of 1 cd.s/m^2^ was presented to the dark-adapted eyes. Three trials were run for the right eye, using a 15 s interstimulus interval, followed by three trials for the left eye. A rod-desensitizing, light adapting field (110 cd/m^2^) was then presented for a period of 3 minutes, after which another drop of methylcellulose was applied to each eye without disturbing the electrodes. Light-adapted, photopic ERG responses were then measured as follows. A total of twenty individual sweeps were recorded from each eye in response to a flash stimulus of 100 cd.s/m^2^, presented alternately to the two eyes using a 1 s interstimulus interval. Upon completion of ERG data acquisition, Puralube Ophthalmic Solution (Dechra Veterinary Products, Overland Park, KS, USA) was applied to each eye, and animals were provided supplemental heat during recovery. The target group size for ERG was four mice per sex per strain and ranged from 1-7 mice per sex per strain. Based on the sexual dimorphism analysis of data from the wildtype control population described below, ERG data were collapsed by sex for each mutant strain tested. The resulting sample size ranged from 5-12 mice per mutant strain. Each strain was tested typically in two separate batches (range of 1–6 batches of mice per strain). The median time interval in which all batches of a given strain were tested was 28 days (range 1–434 days per strain).

At week 17–19, ophthalmic anesthetic eye drops [typically 0.5% Tetracaine HCL Ophthalmic Solution] were applied. Anesthesia was induced using inhaled isoflurane [5% isoflurane (Covetrus North America, Dublin, OH, USA) mixed with 1 L/min of oxygen]. Once the mice were non-responsive to toe-pinch stimulation, blood was collected via the retro-orbital synapse, and mice were euthanized via cervical dislocation. Enucleated eyes were fixed in a solution of methanol: acetic acid: 1x phosphate-buffered saline (prepared fresh in a ratio of 3:1:4) for 24–48 hours, before being processed into paraffin-embedded blocks and sectioned parallel to the optical axis. For each knockout mouse strain, both eyes from at least one male mouse, up to a maximum of eight mice, were sectioned (5 µm) and stained with hematoxylin and eosin. In addition, both eyes from inbred C57BL/6NJ mice (IMSR_JAX:005304, The Jackson Laboratory, Bar Harbor, ME, USA) were collected at regular intervals to generate a pool of representative, contemporaneous wildtype control samples for histopathological comparison (n = 11 mice, 22 eyes). Slides from all mice of strains identified with abnormal ERG were reviewed by a single, experienced, board-certified veterinary anatomic pathologist (Jessica Wong) using an Olympus BX43 microscope (Olympus Corporation, Shinjuku Monolith, 2-3-1 Nishi-Shinjuku, Shinjuku-ku, Tokyo, 163–0914, Japan), and all photomicrographs were captured with an Olympus DP74 camera and cellSens Standard 3.2 software (Olympus Corporation, Shinjuku Monolith, 2-3-1 Nishi-Shinjuku, Shinjuku-ku, Tokyo, 163–0914, Japan) at 0.5 mm from the center of the optic nerve head.

### Quality control and analysis of mouse ERG data

S3 Fig. shows a representative wildtype control waveform for each recording condition used in this study and indicates how the different ERG response components were measured. The scotopic ERG response comprised four components: the negative polarity a-wave, measured from the pre-stimulus baseline to the subsequent negative trough; the positive polarity b-wave, measured from the a-wave trough to the subsequent positive peak; the c-wave, measured from the baseline to the positive peak following the b-wave; and a consistently observed negative polarity component following the c-wave, referred to as the FO-like component, measured from the c-wave peak to the subsequent negative trough. This aligns with a component reported independently under different stimulation and recording conditions, designated as the fast oscillation [51,68,69]. The photopic ERG response comprised two components: an initial negative polarity a-wave followed by a positive polarity b-wave. These were measured as described for the scotopic a- and b-waves.

A multi-step data QC workflow, summarized in S4 Fig, was applied to the ERG data set, which included 18,892 waveforms collected from 4,723 mice. QC began by removing mice with unresolved metadata issues or technical problems observed during data capture. All waveforms were then corrected for offset drift by calculating the mean amplitude of the pre-stimulus levels for all raw data signals (scotopic: 40 data points over 20 ms; photopic 20 datapoints over 10 ms) and adjusting the entire waveform so that the average pre-stimulus baseline was zero. Next, we assessed the consistency of waveforms within the multiple trials conducted for each mouse (3 for scotopic, 20 for photopic; S2 Fig). The mean absolute deviation (MAD) was calculated across these trials and summed over the entire trace for each waveform. When waveforms were very similar across trials, the MAD score was low. Larger MAD scores were obtained when the individual waveforms diverged across trials. We plotted the MAD score distributions separately for scotopic and photopic data and selected the upper 1% of each probability density function distribution as the cutoff. A total of 42 scotopic and 51 photopic waveforms that fell into this range were QC failed due to high inter-trial variability. The individual trials and averaged waveform of the flagged mice were manually reviewed to verify the excessive variability before the mice were QC failed. High MAD scores were typically associated with noise in the ERG signal. No mutant strain had more than two mice fail this step.

The mean ± 3SD of the wildtype control mice was calculated for each parameter, separately for the right and left eye. These limits were applied to the entire dataset, identifying a total of 250 unique mice with extreme values (99 based on scotopic and 174 based on photopic ERG responses). Before removal, the waveforms were manually reviewed to determine the cause of the abnormal reading, which were typically due to poor signal quality. No specific mutant mouse strain was over-represented in these QC failures, although eight strains lost three or four mice from their photopic dataset.

To manage baseline variability over the approximately 3-year period during which these data were collected, we used a windowing approach to select wildtype control mice run contemporaneously with a given knockout line. This soft-window model gave more statistical weight to control animals that were measured nearer in time to the mutant mice [67]. The analysis started with a standard ANOVA comparing all wildtype mice and the mice of one mutant strain for a given eye parameter. The results of this basic analysis were then processed through the soft-windowing algorithm to optimize the size and shape of the window and assign weights to the wildtype mice (between 0 and 1). Mice with weights close to zero contributed virtually nothing to the analysis, while mice with weights close to one formed the effective control group. Optimization ensured that enough mice were retained to provide adequate power for testing while minimizing the variability of the wildtype data. The model optimized each analysis independently, so the effective control group varied for the same strain across eye parameters. The window shapes could vary considerably across mutant strains; for example, if all the mice for a knockout strain were collected close in time, the window would be relatively narrow. However, if mice were collected in cohorts well separated in time, the window would be quite broad or even bimodal.

Once the window was optimized, the OpenStats [70] software package was used to recalculate the ANOVA using the weighted control data to provide the *p*-value and the effect size. Effect size was recorded as the percentage change from wildtype using the coefficients from the linear model (Y = B_0_ + B_1_*genotype; effect size = 100*B_1_/B_0_) [71].

The concordance of the ERG response was examined at three levels. First, within eyes across trials using the MAD analysis described above. Next, concordance was evaluated between eyes of a given animal, and finally, across all the animals within a given knockout strain. Each eye parameter for the knockout strain was independently tested statistically against the corresponding wildtype parameter as described above, and then the results of both eyes were assessed together. We derived a combined mean for the percentage changes in both eyes (henceforth termed mean-effect percentage) and the associated *p*-value, to ensure concordance between eyes. The *p*-value threshold profile required to identify a strain as statistically significant was *p* ≤ 0.01 for each eye and *p* ≤ 0.0001 for data combined between the two eyes. This stringent combination of thresholds was designed to provide conservative estimates of biologically significant effects. Only parameters with statistical significance agreement of both eyes (concordance) were considered to be hits.

The R based code, the data with windowing information for the control mice, as well as the output of all analyses, including the initial and final optimized statistical models, can be found at the Zenodo repository (https://zenodo.org/records/16944276).

### Assessment of mouse ERG data for sexual dimorphism

Data from wildtype control mice were assessed for sexual dimorphism for both scotopic (n = 316 males; n = 315 females) and photopic (n = 309 males; n = 315 females) measures using Welch’s t-tests. Data were compared by sex for each eye separately, and significance was determined using a Bonferroni-corrected threshold of 0.004 (corrected for 12 tests; six for each eye). Our extremely large sample sizes (>300) provide strong power to identify any existing sex differences, allowing us to confidently conclude that for a-wave and b-wave measures, under both scotopic and photopic conditions, there is no sexual dimorphism in the mouse ERG response. Although the right eye measures of c-wave and FO-like wave indicated small differences between the sexes, none of the left eye measures were significantly different. As it is unlikely that sexual dimorphism would occur only in one eye, we conclude that dimorphism was not apparent in the wildtype control ERG data. S5 Fig shows all the values for each measure, with ranges indicated by error bars.

### Counting of mouse photoreceptor nuclei

Whole slide images (WSIs) of H&E-stained histological slides containing 10–21 ocular sections were obtained with a NanoZoomer C9600 2.0-HT or C13239 S210 slide scanner (Hamamatsu, Bridgewater, NJ, USA) at 20x or 40x resolution and hosted on an OMERO [72] server. A total of 204 slide scans from 30 knockout strains and C57BL/6NJ control mice, all examined at 17–19 weeks of age, were imported into a QuPath 0.5.0-arm64 [73] project using the OMERO 0.4.0 extension. WSIs were reviewed to identify in-focus sections containing the optic nerve head as a landmark, which were marked with a rectangular annotation that included the full retina. If the optic nerve head was not encountered (12 slides) or if all sections were damaged (1 slide), the slide was excluded from further analysis. Sections containing the optic nerve head and a gap in the iris corresponding to the pupil were considered suitably oriented for accurate counting; slides of oblique sections that included a continuous contour of pigmented cells (iris, ciliary margin, or RPE) were excluded (24 slides). Rectangular annotations from the included 167 slides were batch-exported at full resolution from QuPath in.jpg format; sections imaged at 40x were downsized 2-fold in Fiji [74]. The.jpg images were analyzed using a custom Fiji macro for manual counting, as described previously [64]. The macro draws rotated rectangles with a fixed width parallel to the outer retinal perimeter at regular intervals from the center of the optic nerve head. Objects within each rectangle are counted manually with the multipoint tool, the annotation is saved in the ROI Manager, and counts are reported for further analysis. For this project, photoreceptor nuclei localized within or near the ONL in 25-µm wide rectangles centered 0.5 mm from the optic nerve head center were counted (S7a Fig). Nuclei intersecting the rectangle boundary were excluded. Counts from both sides of the optic nerve head were averaged from a single section of each eye analyzed. If both eyes from a single mouse were available, the values from both eyes were averaged further. Photoreceptor nuclei counts from knockout cohorts of n ≥ 3 were compared with C57BL/6NJ wildtype control counts (n = 11 mice, 21 eyes) by one-way ANOVA and Dunnett’s multiple comparison test in GraphPad Prism 10.2.3 (Dotmatics, Boston, MA, USA) using a significance threshold of *p *= 0.05.

### Screening ERG candidate genes in unsolved IRD patients

We screened approximately 800 patients diagnosed with inherited retinal disease, but no conclusive molecular diagnosis after exome or genome sequencing. Each patient underwent a standard clinical exam, including patient and family history, dilated fundus exam, best-corrected visual acuity, Goldmann visual field testing, and full-field ERG [75].

The impact of the missense variant p.Gln397Arg was predicted using the CADD Phred3 and pop-EVE4 algorithms [37,38]. The variant was finally classified according to guidelines from the American College of Medical Genetics and Genomics [40].

## Supporting information

S1 Fig Schematic of the IMPC mouse phenotyping pipeline detailing weekly testing at The Jackson Laboratory. Typically, eight male and eight female mutant mice were assessed per assay for each single-gene knockout mouse strain. Exceptions from the norm, such as electroretinography was performed on four males and four females per strain, are noted in blue text. Additionally, five male and five female wildtype C57BL/6NJ control mice were tested each week.
(PDF)

S2 Fig Diagram of the ERG protocol used here. After overnight dark adaptation and initial setup, amplifier output quality was previewed. Once stable and following a 10 s baseline recording, six scotopic trials (three per eye) were conducted with bright (1 cd.s/m^2^) flashes presented unilaterally at 15 s interstimulus intervals, recording responses separately. Trials were run for the right eye first, followed by the left eye. A steady rod-desensitizing adapting field (110 cd/m^2^) was presented for 3 minutes. After a 6 s baseline recording, forty photopic trials (20 per eye), alternating between right and left eyes with the unstimulated eye serving as a control, were conducted with the flashes (100 cd.s/m^2^) superimposed on the adapting field. The interstimulus interval was 1 s, and each response was stored separately.
(PDF)

S3 Fig Representative wildtype control waveform for each recording condition used in this study (black lines), annotated with ERG response component measurements (blue arrows), and the temporal windows within which they were measured (grey bars). The a-wave was measured from baseline to the subsequent negative trough for both scotopic (left panel) and photopic (right panel) waveforms. The b-wave was measured from the a-wave trough to the positive peak for both scotopic (left panel) and photopic (right panel) waveforms. The scotopic c-wave was measured from the baseline to the peak that followed the b-wave (middle panel), and the FO-like component was measured from the c-wave peak to the subsequent negative trough (middle panel).
(PDF)

S4 Fig Overview of the multi-step data QC workflow applied in this study, including generic QC, offset correction, a two-phase statistical outlier assessment, and conventional manual review by a domain expert. (PDF)

S5 Fig Assessment of sexual dimorphism in wildtype control mice for scotopic (n = 316 males; n = 315 females) and photopic (n = 309 males; n = 315 females) amplitude measures. Data were compared by sex for each eye separately, with significance determined using a Bonferroni-corrected threshold of 0.004 (indicated by an orange #). Error bars indicate minimum and maximum ranges for each measure.
(PDF)

S6 Fig ERG waveforms for all 30 strains, listed alphabetically by gene symbol, identified with a significant change in amplitude of at least one ERG response component. Each strain is represented by three panels: the left panel shows the first 200 ms of the scotopic ERG response, focusing on the a- and b-waves; the middle panel presents the entire 5 s scotopic recording epoch, highlighting the c-wave and FO-like component; the right panel displays the photopic ERG response. In each panel, the pale blue shaded area indicates the ± 1 SD range around the mean for all wildtype traces (scotopic n = 631, photopic n = 624), and the solid blue line represents the wildtype mean. Gray traces represent the mean responses of both eyes for individual mutant mice, with the solid black line indicating the mean response for the mutant strain.
(PDF)

S7 Fig Mean photoreceptor nuclei count of ERG mutant strains. (a) Nuclei were counted within 25-µm wide segments of the ONL centered 0.5 mm from the center of the optic nerve head. (b) Counts on both sides of the optic nerve head and from one or both eyes of each mouse examined were averaged. Plotted values indicate mean ± SD. The number of mice and eyes assessed for each strain is annotated at the bottom of panel b. One-way ANOVA indicated a statistically significant effect of strain on photoreceptor nuclei count: F(17, 62) = 57.6; *p *= 1.9 × 10^−31^. Significant *p* values from Dunnett’s multiple comparison test against C57BL/6NJ (wildtype) samples are indicated. The 95% confidence interval for wildtype controls is indicated (light blue shading).
(PDF)

S1 Table Metadata and results of statistical analysis to detect genotype effects within ERG responses from 530 single-gene knockout mouse strains. Significant *p*-values (*p* ≤ 0.01 for each measure tested and *p* ≤ 0.0001 for the combined left eye*right eye concordance measure) are highlighted in yellow. Gray cells indicate insufficient data for statistical analysis after rigorous QC and sample size threshold application.
(XLSX)

S2 Table Metadata and results of statistical analysis to detect sexual dimorphism within ERG responses from 124 single-gene knockout mouse strains (scotopic amplitude) and 117 single-gene knockout mouse strains (photopic amplitude). Significant *p*-values (*p* ≤ 0.01 for each measure tested and *p* ≤ 0.0001 for the combined left eye*right eye concordance measure) are highlighted in yellow. Gray cells indicate insufficient data for statistical analysis after rigorous QC and sample size threshold application.
(XLSX)
