# Supplementary figures and images for: Identifying genetic determinants of outer retinal function in mice using a large-scale gene-targeted screen
Source: PLoS Genet. 2025 Sep 29;21(9):e1011886. doi: 10.1371/journal.pgen.1011886 (PMC12503315; doi:10.1371/journal.pgen.1011886)

Supplementary Figure 1

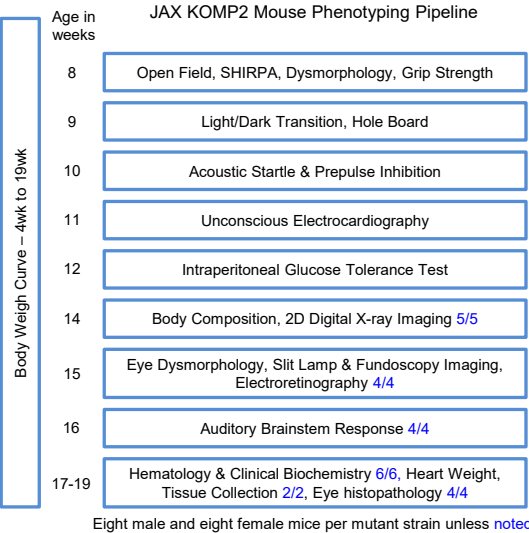

Supplement: S1 Fig — Typically, eight male and eight female mutant mice were assessed per assay for each single-gene knockout mouse strain. Exceptions from the norm, such as electroretinography was performed on four males and four females per strain, are noted in blue text. Additionally, five male and five female wildtype C57BL/6NJ control mice were tested each week. (PDF) [file pgen.1011886.s001.pdf]

### Scotopic

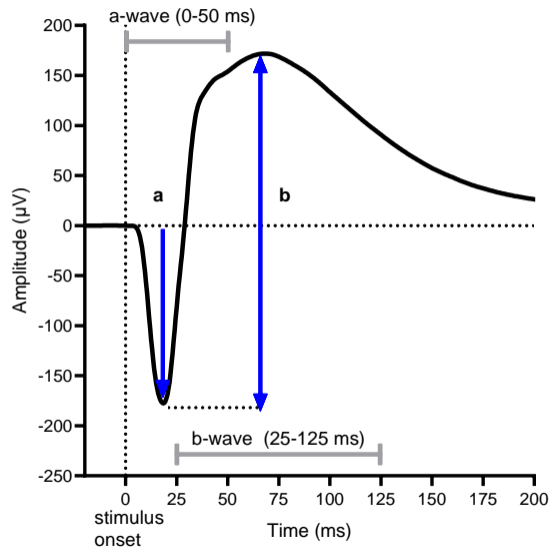

### Scotopic

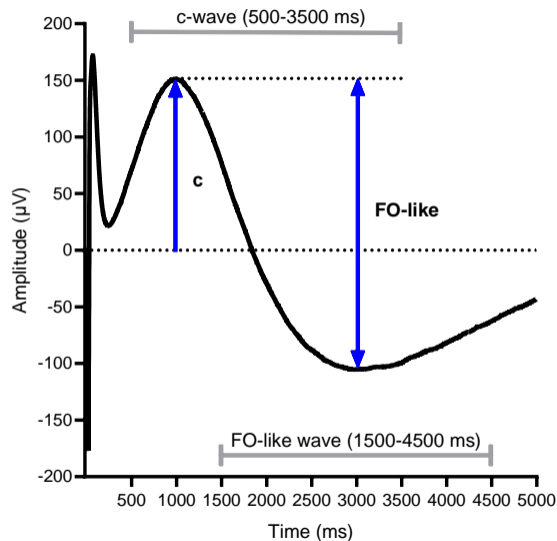

### Photopic

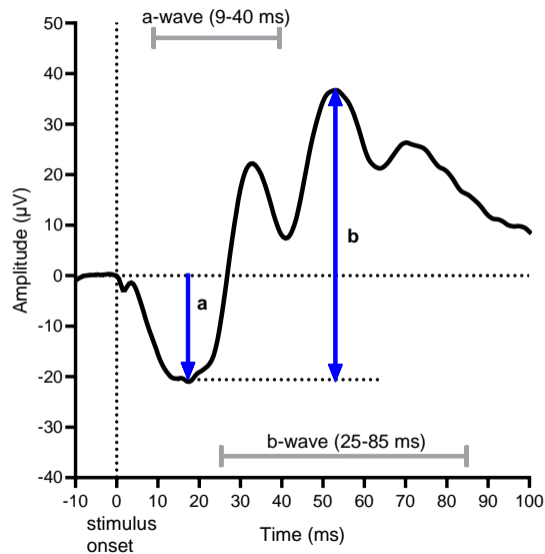

Supplement: S3 Fig — The a-wave was measured from baseline to the subsequent negative trough for both scotopic (left panel) and photopic (right panel) waveforms. The b-wave was measured from the a-wave trough to the positive peak for both scotopic (left panel) and photopic (right panel) waveforms. The scotopic c-wave was measured from the baseline to the peak that followed the b-wave (middle panel), and the FO-like component was measured from the c-wave peak to the subsequent negative trough (middle panel). (PDF) [file pgen.1011886.s003.pdf]

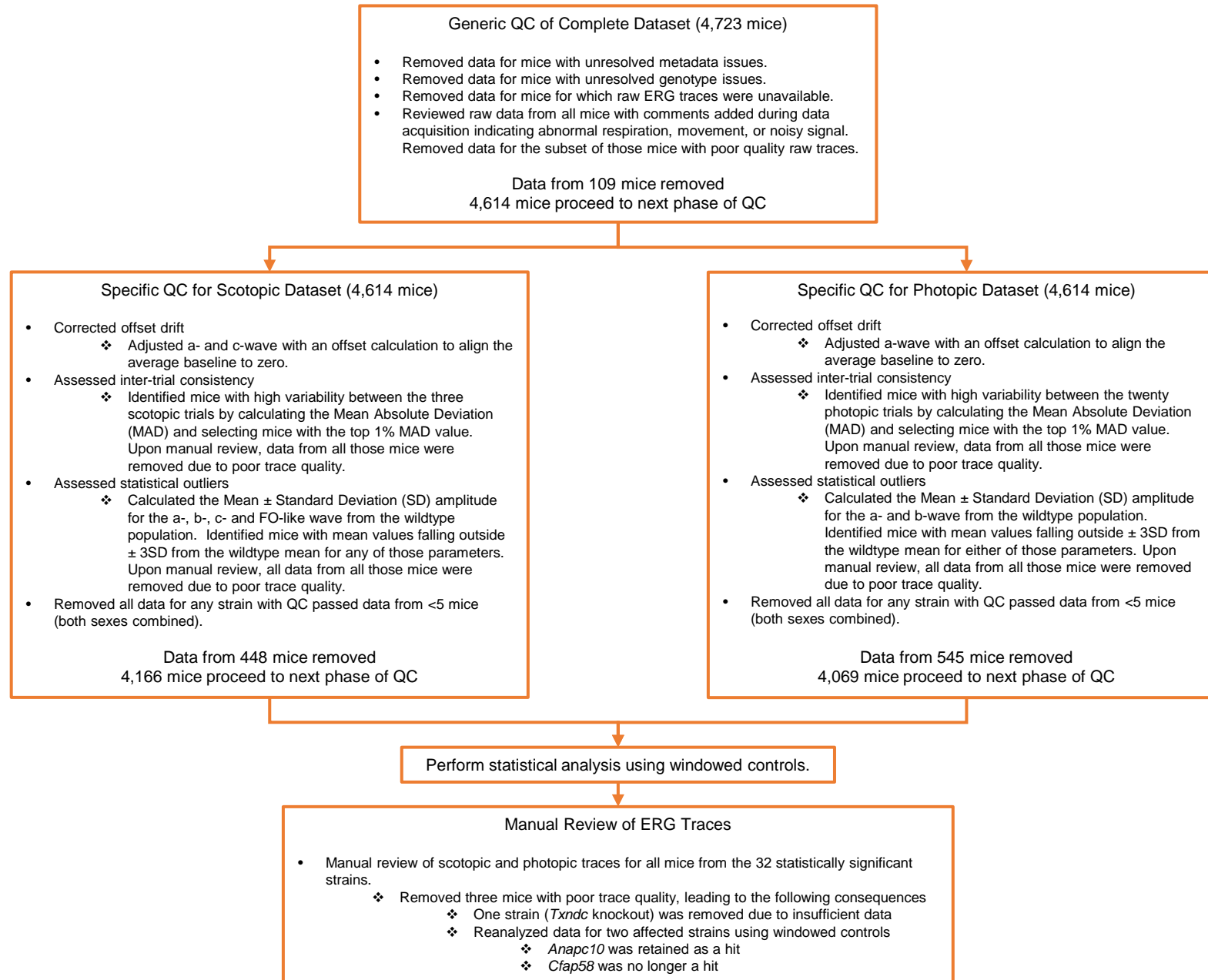

Supplement: S4 Fig — (PDF) [file pgen.1011886.s004.pdf]

## Scotopic

## Photopic

Left  
Eye

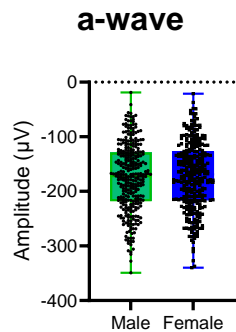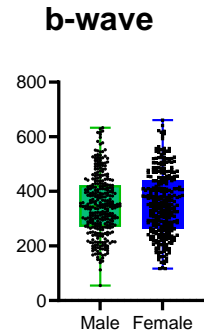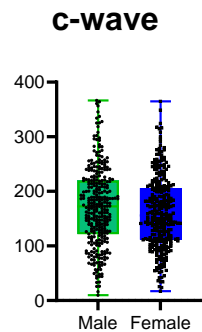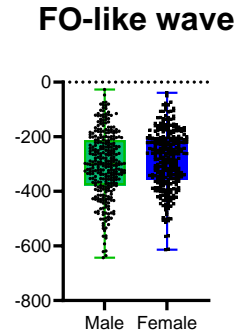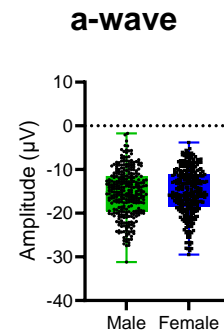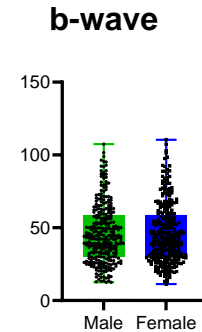

Right  
Eye

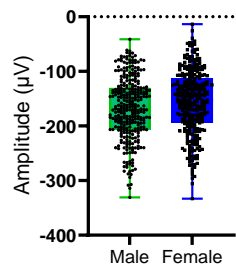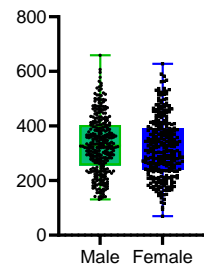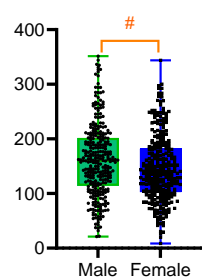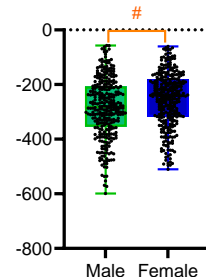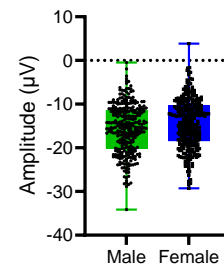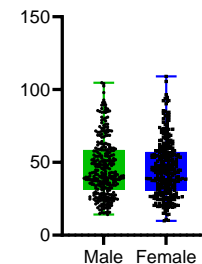

Supplement: S5 Fig — Data were compared by sex for each eye separately, with significance determined using a Bonferroni-corrected threshold of 0.004 (indicated by an orange #). Error bars indicate minimum and maximum ranges for each measure. (PDF) [file pgen.1011886.s005.pdf]

**A**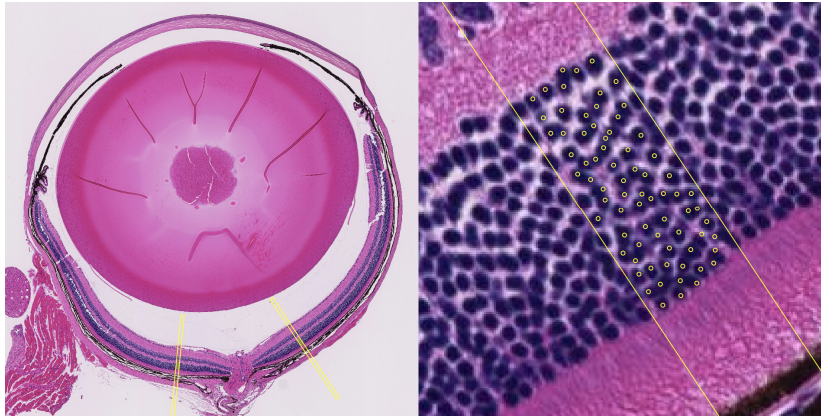**B**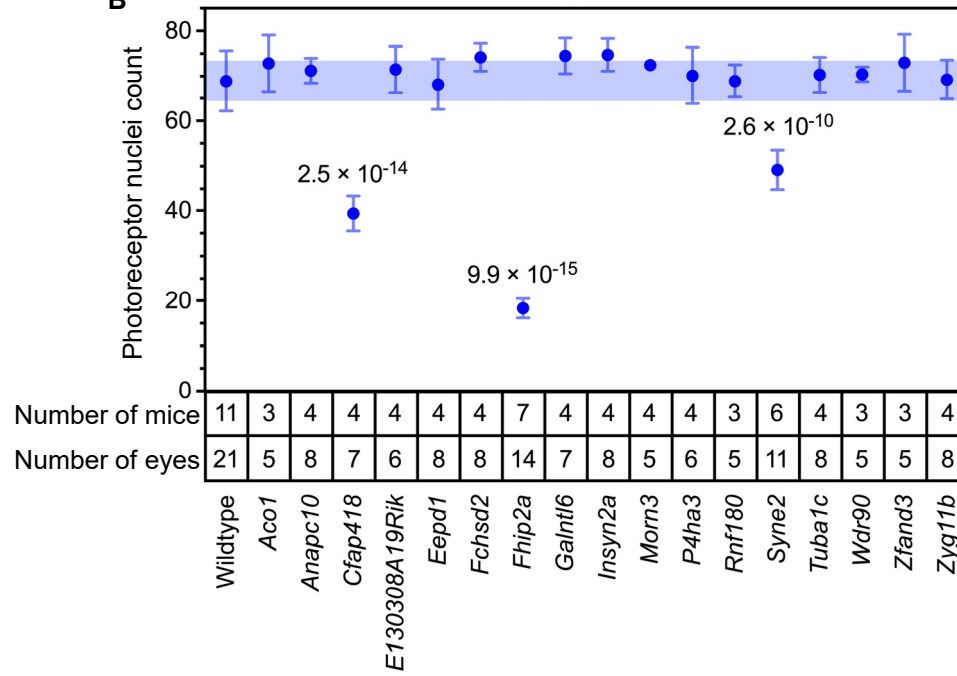

Supplement: S7 Fig — (a) Nuclei were counted within 25-µm wide segments of the ONL centered 0.5 mm from the center of the optic nerve head. (b) Counts on both sides of the optic nerve head and from one or both eyes of each mouse examined were averaged. Plotted values indicate mean ± SD. The number of mice and eyes assessed for each strain is annotated at the bottom of panel b. One-way ANOVA indicated a statistically significant effect of strain on photoreceptor nuclei count: F(17, 62) = 57.6; p = 1.9 × 10^−31^. Significant p values from Dunnett’s multiple comparison test against C57BL/6NJ (wildtype) samples are indicated. The 95% confidence interval for wildtype controls is indicated (light blue shading). (PDF) [file pgen.1011886.s007.pdf]
