# Supplementary material for: Identifying genetic determinants of outer retinal function in mice using a large-scale gene-targeted screen
Source: PLoS Genet. 2025 Sep 29;21(9):e1011886. doi: 10.1371/journal.pgen.1011886 (PMC12503315; doi:10.1371/journal.pgen.1011886)
Supplement: S2 Fig — After overnight dark adaptation and initial setup, amplifier output quality was previewed. Once stable and following a 10 s baseline recording, six scotopic trials (three per eye) were conducted with bright (1 cd.s/m2) flashes presented unilaterally at 15 s interstimulus intervals, recording responses separately. Trials were run for the right eye first, followed by the left eye. A steady rod-desensitizing adapting field (110 cd/m2) was presented for 3 minutes. After a 6 s baseline recording, forty photopic trials (20 per eye), alternating between right and left eyes with the unstimulated eye serving as a control, were conducted with the flashes (100 cd.s/m2) superimposed on the adapting field. The interstimulus interval was 1 s, and each response was stored separately. (PDF) [file pgen.1011886.s002.pdf]

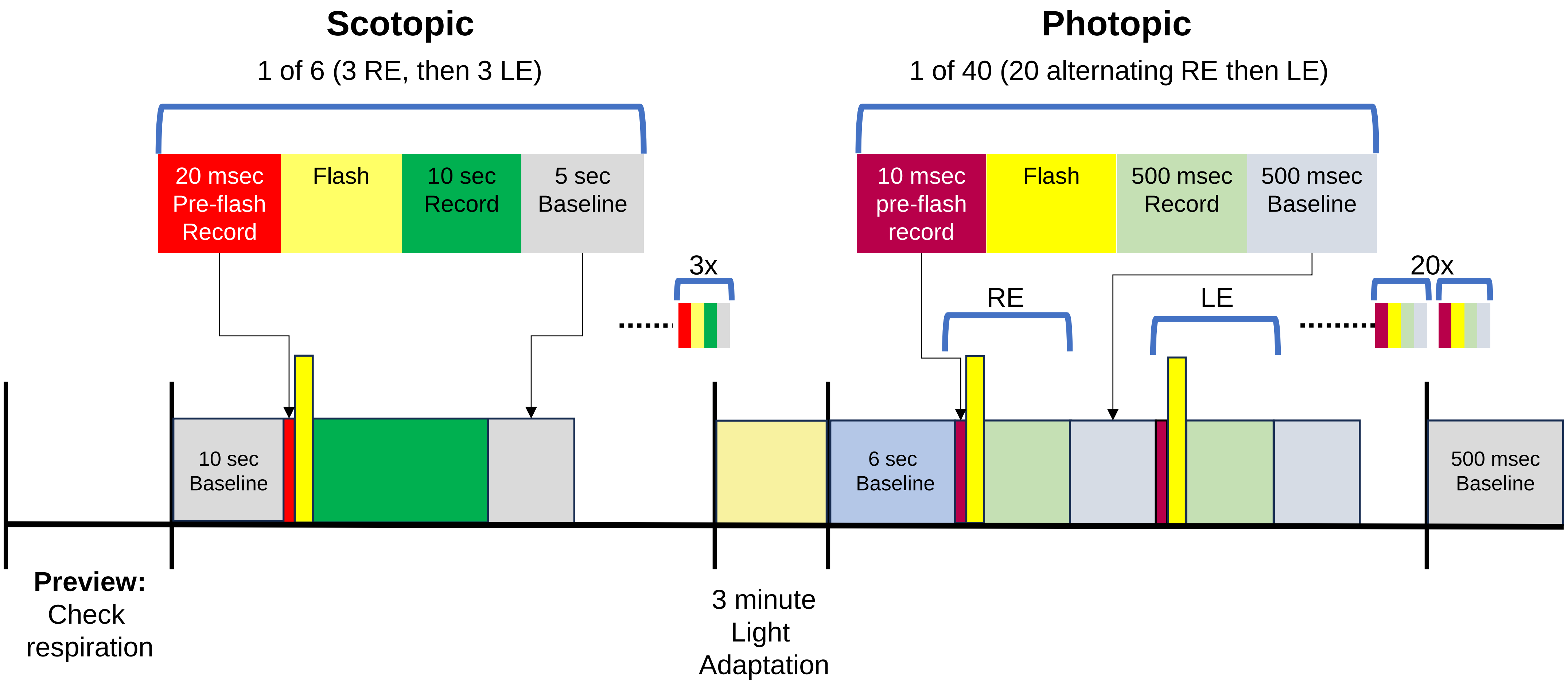

Scotopic
1 of 6 (3 RE, then 3 LE)
20 msec Pre-flash Record
Flash
10 sec Record
5 sec Baseline
3x
Photopic
1 of 40 (20 alternating RE then LE)
10 msec pre-flash record
Flash
500 msec Record
500 msec Baseline
20x
RE
LE
10 sec Baseline
6 sec Baseline
500 msec Baseline
Preview: Check respiration
3 minute Light Adaptation
