# Supplementary material for: Identifying genetic determinants of outer retinal function in mice using a large-scale gene-targeted screen
Source: PLoS Genet. 2025 Sep 29;21(9):e1011886. doi: 10.1371/journal.pgen.1011886 (PMC12503315; doi:10.1371/journal.pgen.1011886)
Supplement: S6 Fig — Each strain is represented by three panels: the left panel shows the first 200 ms of the scotopic ERG response, focusing on the a- and b-waves; the middle panel presents the entire 5 s scotopic recording epoch, highlighting the c-wave and FO-like component; the right panel displays the photopic ERG response. In each panel, the pale blue shaded area indicates the ± 1 SD range around the mean for all wildtype traces (scotopic n = 631, photopic n = 624), and the solid blue line represents the wildtype mean. Gray traces represent the mean responses of both eyes for individual mutant mice, with the solid black line indicating the mean response for the mutant strain. (PDF) [file pgen.1011886.s006.pdf]

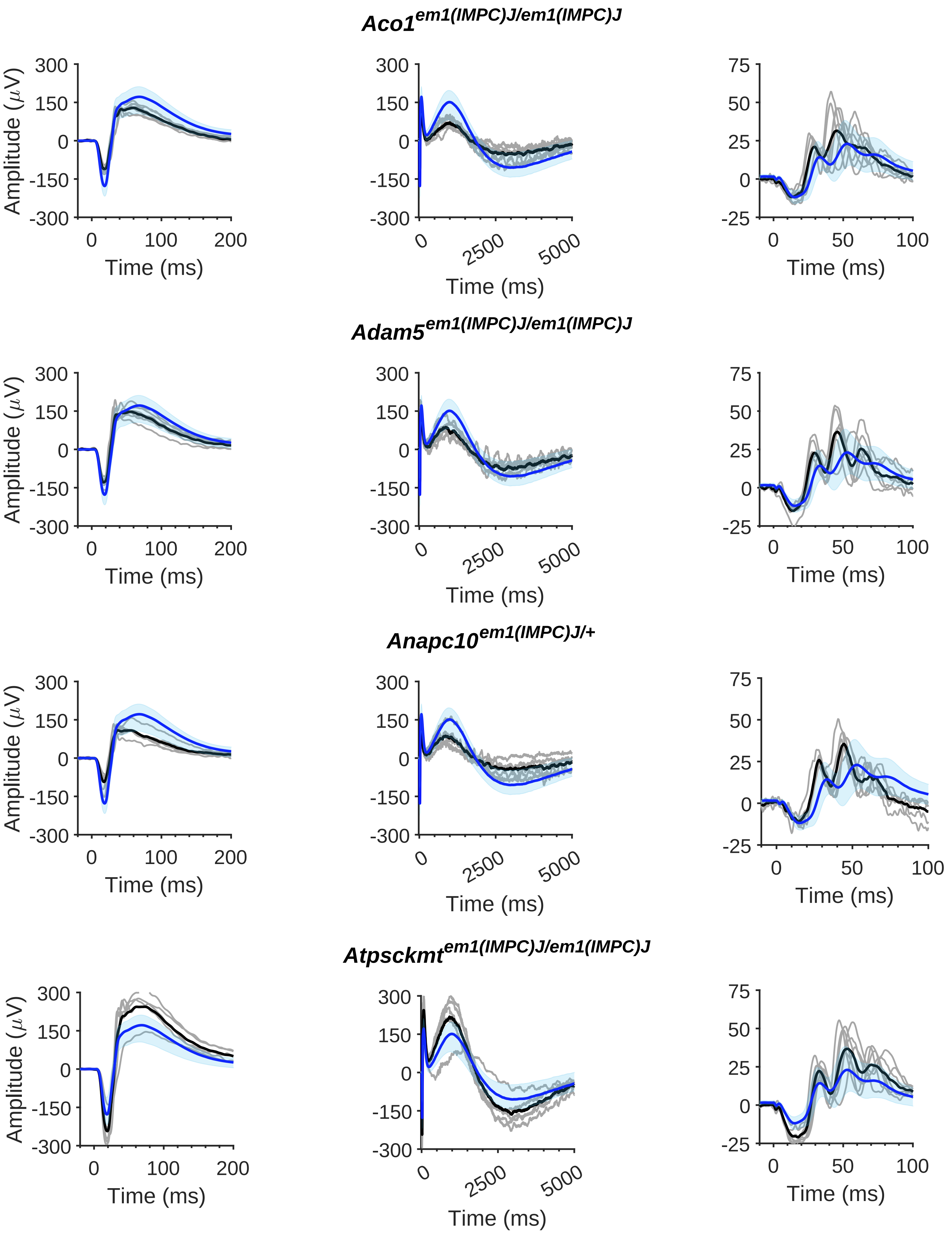

Aco1em1(IMPC)J/em1(IMPC)J
Adam5em1(IMPC)J/em1(IMPC)J
Anapc10em1(IMPC)J/+
Atpsckmtem1(IMPC)J/em1(IMPC)J
Amplitude (μV)
Time (ms)

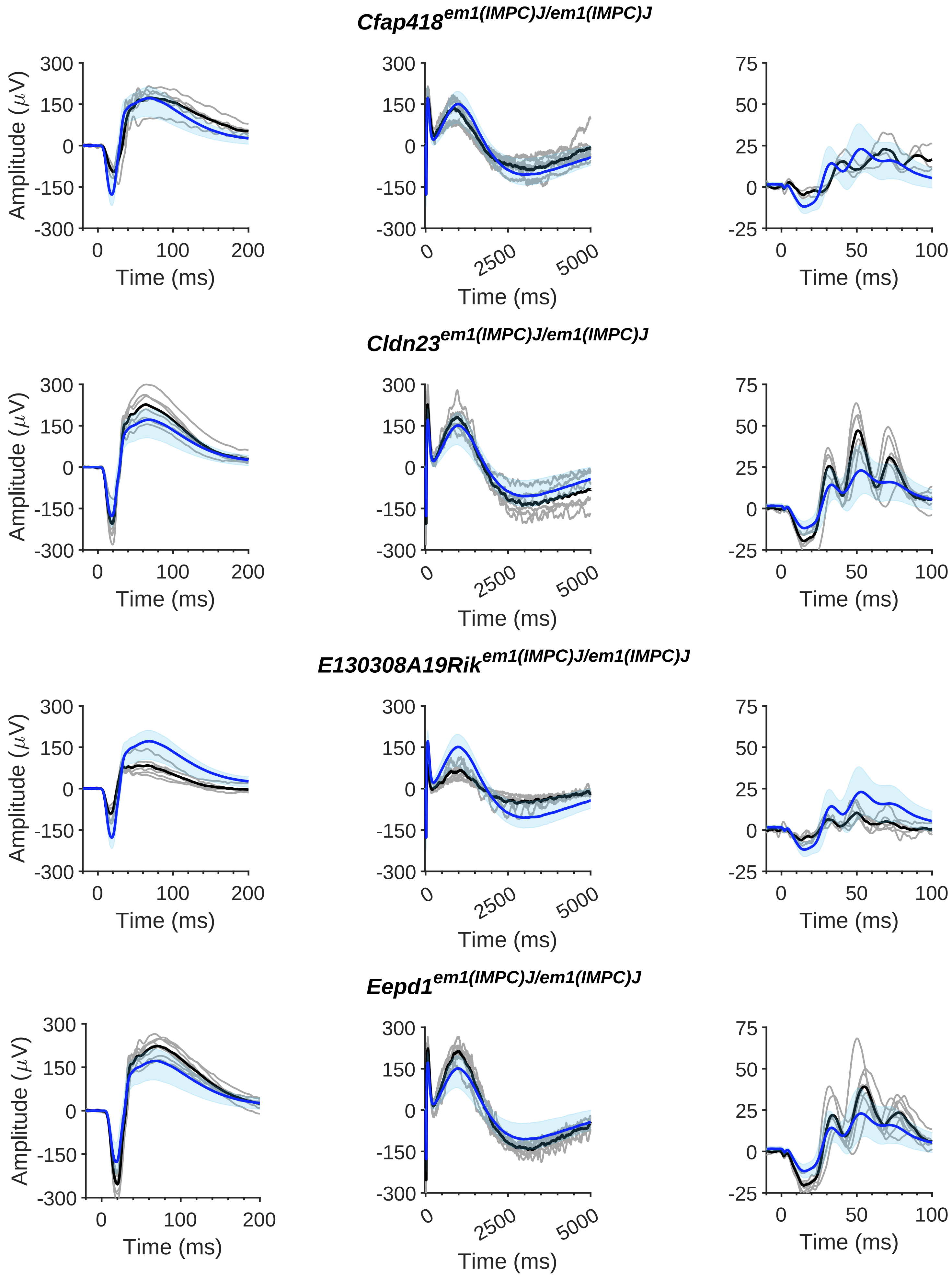

$Cfap418^{em1(IMPC)J/em1(IMPC)J}$
$Cldn23^{em1(IMPC)J/em1(IMPC)J}$
$E130308A19Rik^{em1(IMPC)J/em1(IMPC)J}$
$Eepd1^{em1(IMPC)J/em1(IMPC)J}$
Amplitude (μV)
Time (ms)

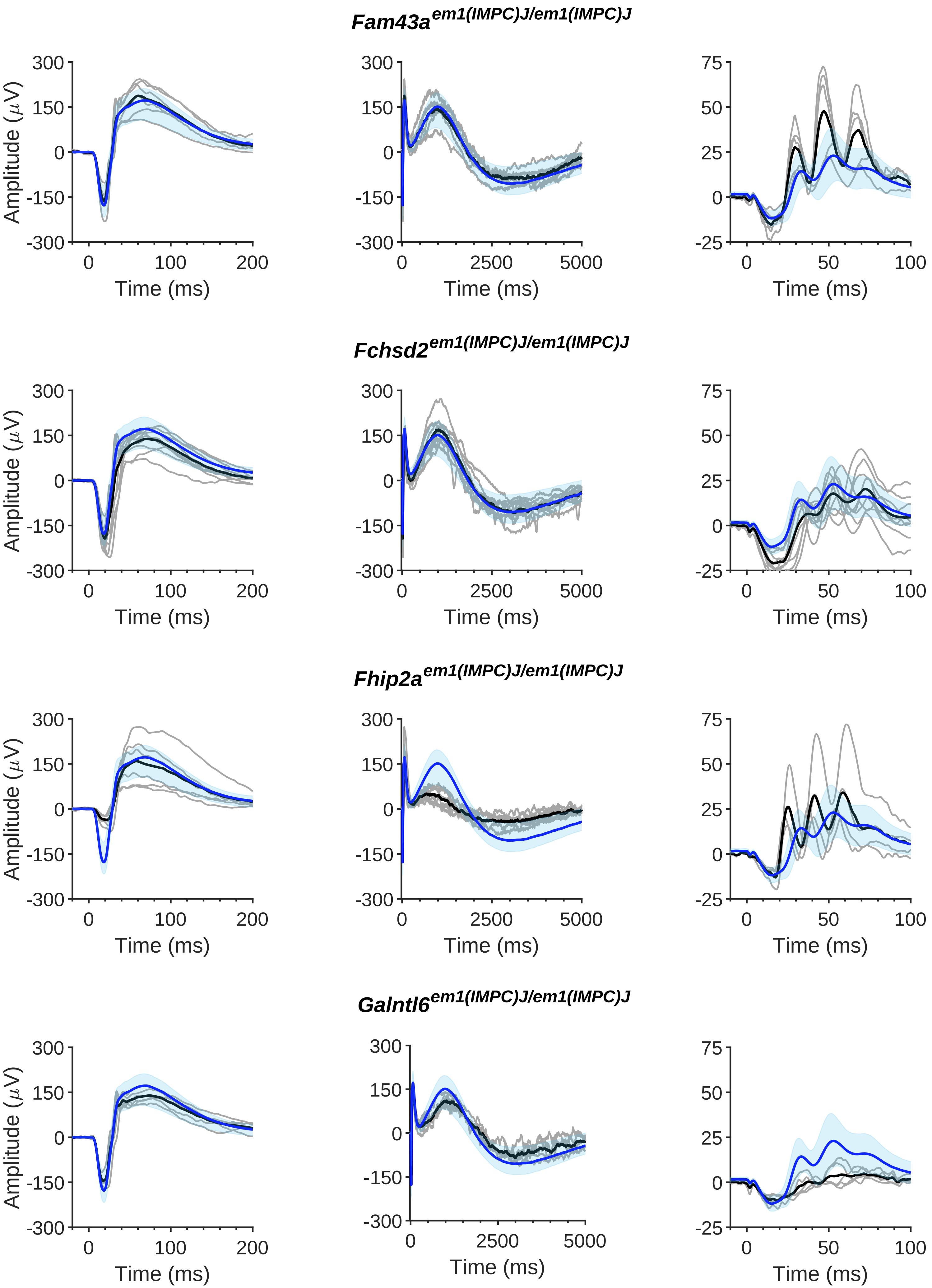

Fam43a em1(IMPC)J/em1(IMPC)J
Fchsd2 em1(IMPC)J/em1(IMPC)J
Fhip2a em1(IMPC)J/em1(IMPC)J
Galntl6 em1(IMPC)J/em1(IMPC)J
Amplitude (μV)
Time (ms)
300
150
0
-150
-300
75
50
25
-25
100
200
2500
5000

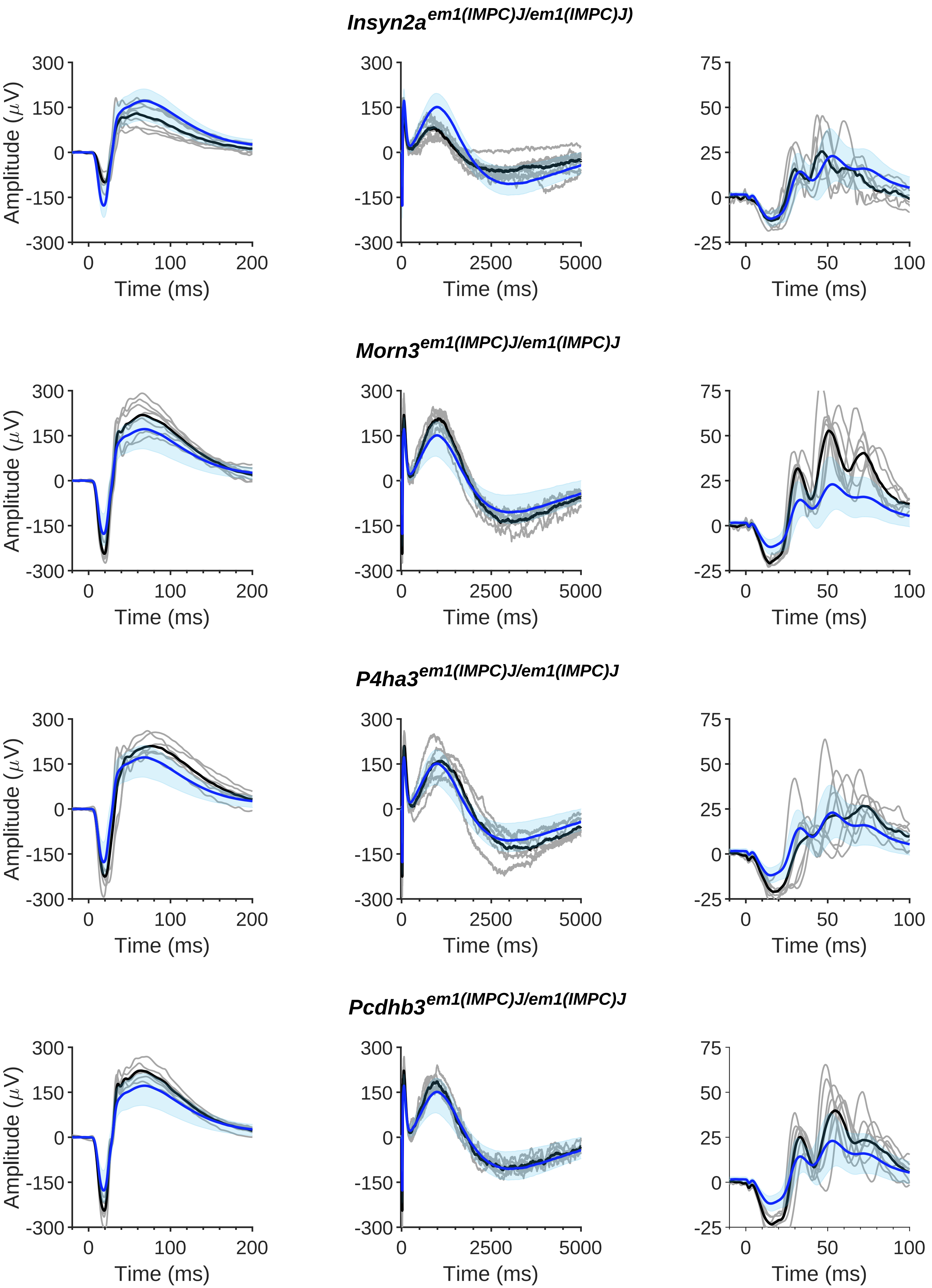

Insyn2a em1(IMPC)J/em1(IMPC)J
Morn3 em1(IMPC)J/em1(IMPC)J
P4ha3 em1(IMPC)J/em1(IMPC)J
Pcdhb3 em1(IMPC)J/em1(IMPC)J
Amplitude (µV)
Time (ms)
300
150
0
-150
-300
75
50
25
0
-25
0
100
200
2500
5000
50

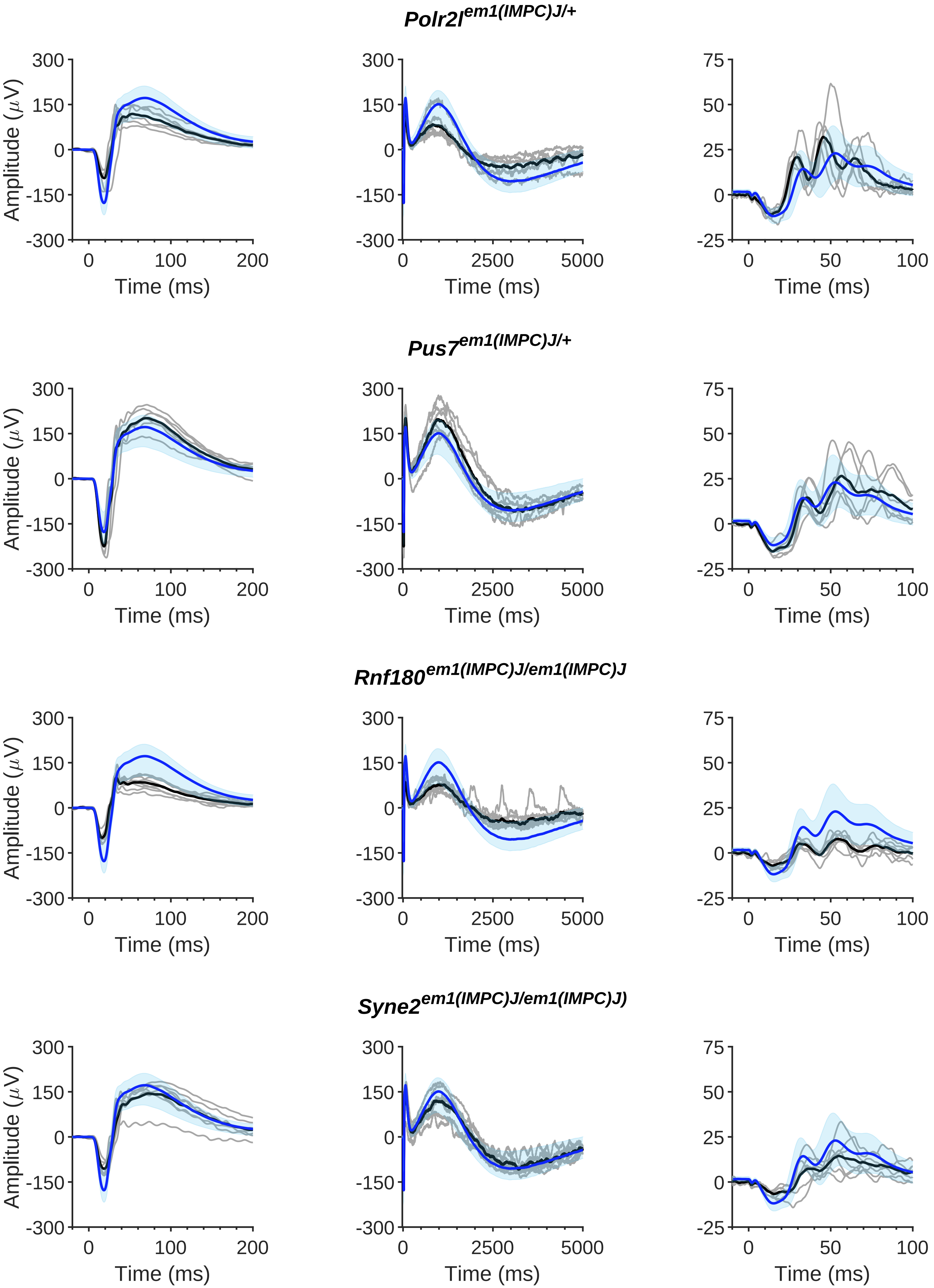

Polr2lem1(IMPC)J/+
Pus7em1(IMPC)J/+
Rnf180em1(IMPC)J/em1(IMPC)J
Syne2em1(IMPC)J/em1(IMPC)J)
Amplitude (μV)
Time (ms)
300
150
0
-150
-300
75
50
25
0
-25
0
100
200
2500
5000
50

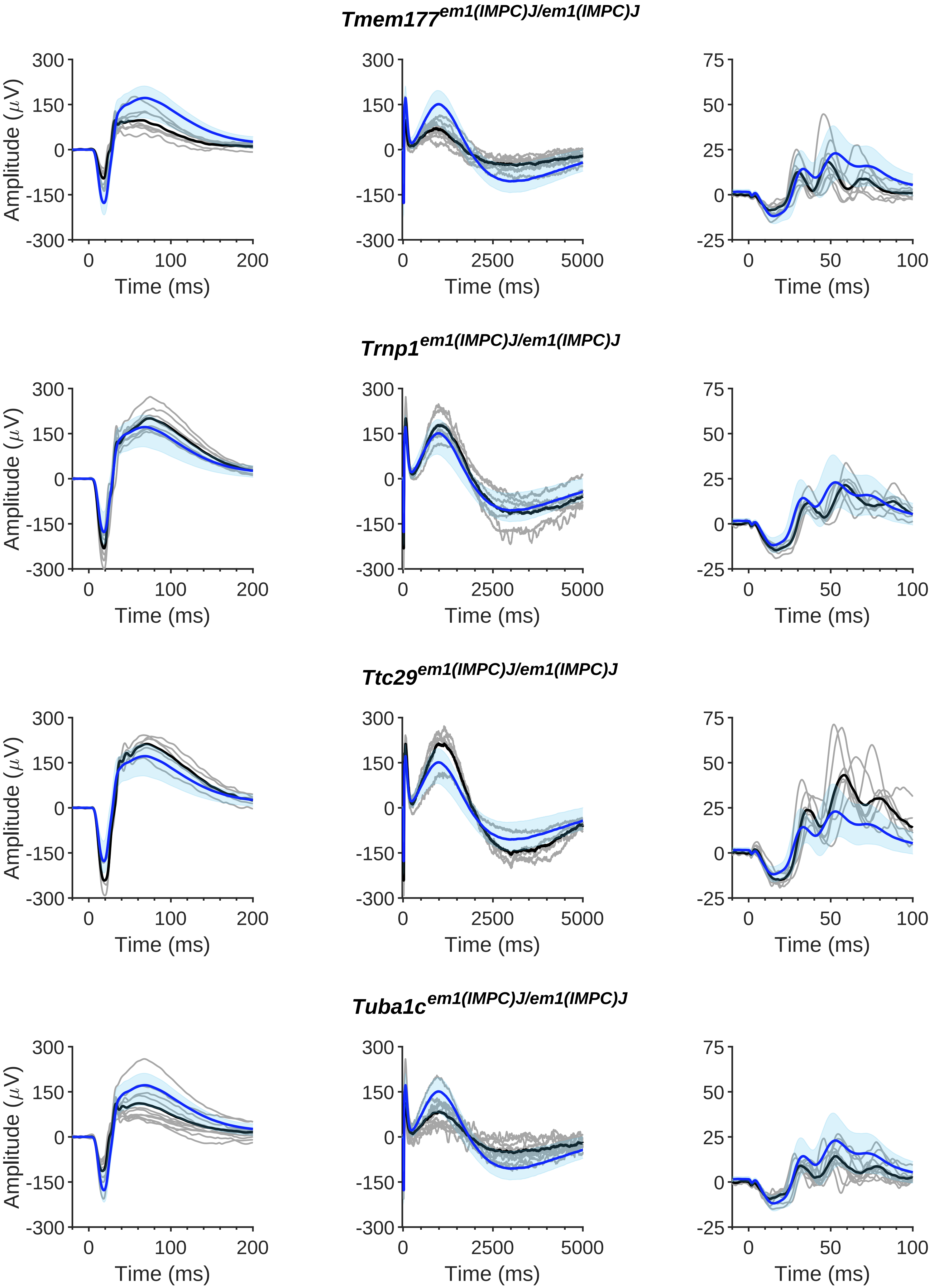

Tmem177em1(IMPC)J/em1(IMPC)J
Trnp1em1(IMPC)J/em1(IMPC)J
Ttc29em1(IMPC)J/em1(IMPC)J
Tuba1cem1(IMPC)J/em1(IMPC)J
Amplitude (μV)
Time (ms)
300
150
0
-150
-300
75
50
25
-25
100
200
2500
5000

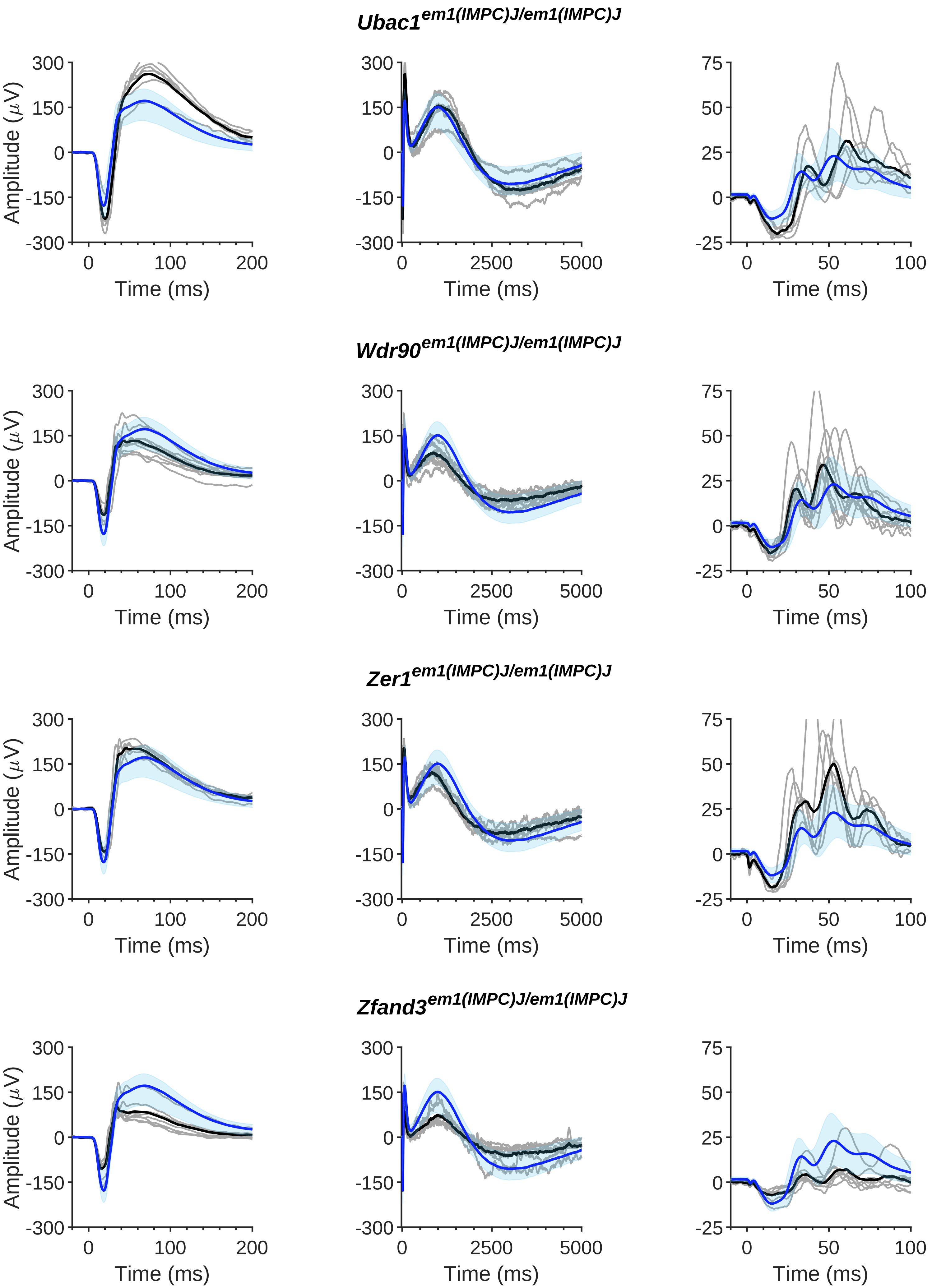

$Ubac1^{em1(IMPC)J/em1(IMPC)J}$
$Wdr90^{em1(IMPC)J/em1(IMPC)J}$
$Zer1^{em1(IMPC)J/em1(IMPC)J}$
$Zfand3^{em1(IMPC)J/em1(IMPC)J}$
Amplitude (μV)
Time (ms)
300
150
0
-150
-300
75
50
25
0
-25
0
100
200
2500
5000
50

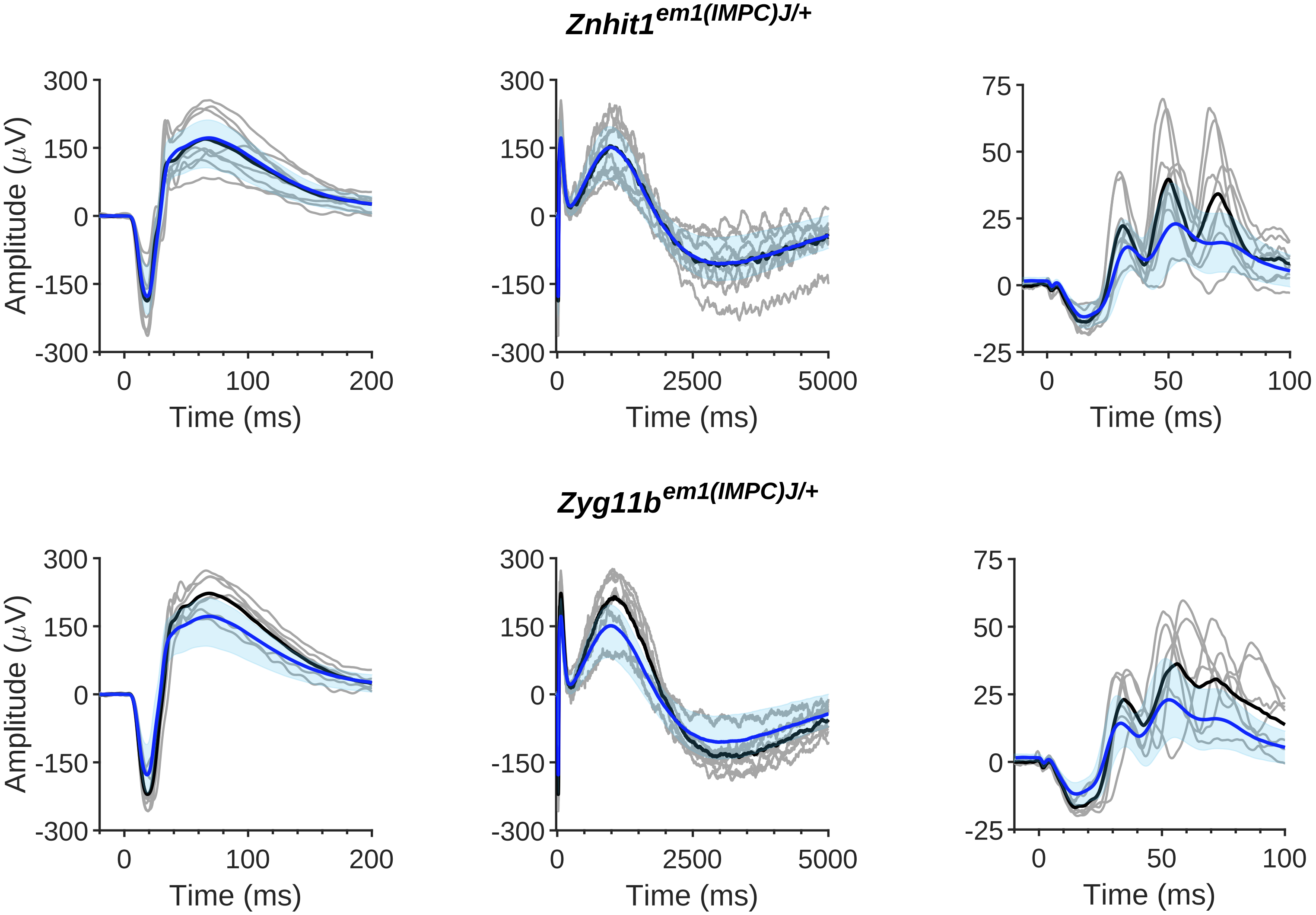

Znhit1em1(IMPC)J/+
Zyg11bem1(IMPC)J/+
Amplitude (μV)
Time (ms)
300
150
0
-150
-300
75
50
25
0
-25
0
100
200
2500
5000
50
